# PET-Trackable Carborane-Conjugated Star-Shaped Poly(2-Oxazoline) Scaffold with High Boron Payload and Improved Aqueous Solubility for Potential Application in Boron Neutron Capture Therapy

**DOI:** 10.1021/acs.biomac.6c00871

**Published:** 2026-07-14

**Authors:** Yuquan Chi, Diana Barakhtii, Alessia Centanni, Vladimir Aseyev, Filip S. Ekholm, Mirkka Sarparanta, Robert Luxenhofer, Surachet Imlimthan

**Affiliations:** Department of Chemistry, 3835 University of Helsinki, FI-00014 Helsinki, Finland

## Abstract

Boron neutron capture therapy (BNCT) requires high, tumor-selective boron delivery, yet carboranes, boron-rich BNCT building blocks, often suffer from poor aqueous solubility and suboptimal *in vivo* behavior despite exceptional boron density and stability. We report a modular star-shaped poly­(2-oxazoline) (POx) scaffold that carries a high carborane payload and can be followed by positron emission tomography (PET). An alkyne-bearing POx was conjugated with azidopropyl *meta*-carborane, yielding PBMM-*m*CB_20_-EIP that remained highly water-soluble (225 g/L, ∼26 g/L of boron, 20 carboranes/star). PBMM-*m*CB_20_-EIP exhibited good cytocompatibility in CAL 27 and FaDu head-and-neck cancer cells and showed higher cell-associated boron concentration than the clinical agent sodium borocaptate. A DOTA-modified analogue was prepared and radiolabeled with gallium-68 for PET imaging. In healthy mice, gallium-68-labeled DOTA-PBMM-*m*CB_20_ revealed substantial blood-pool activity (17%ID/g) at 90 min, predominant renal clearance, and low background uptake, providing a baseline for further macromolecular engineering of this carrier and irradiation-timing optimization in tumor models.

## Graphical abstract

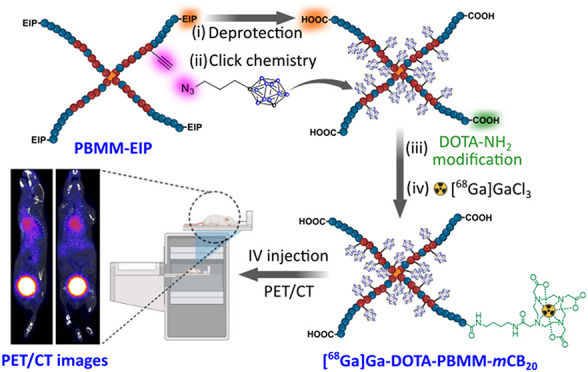

## Introduction

Boron neutron capture therapy (BNCT) is a targeted radiotherapy in which a nonradioactive boron-containing agent is administered prior to neutron irradiation.^1^ During neutron irradiation, epithermal neutrons thermalize in tissue and are captured by the accumulated boron, producing high-linear energy transfer (LET) α particles (^4^He, ≈150 keV/μm) and recoiling lithium-7 nuclei (^7^Li, ≈175 keV/μm) with micrometer-scale path lengths, i.e., on the order of a single cell diameter (∼5–10 μm).^2^ This highly localized energy deposition can selectively damage boron-containing tumor cells while sparing surrounding healthy tissues.^3–5^ Despite the appeal of this mechanism, BNCT outcome has been severely constrained by the ability to deliver sufficiently high boron levels to tumors selectively with favorable pharmacokinetics.

Currently, boron delivery in clinical BNCT has relied primarily on two low-molecular-weight agents, including L-*p*-boronophenylalanine (BPA) and sodium borocaptate (BSH), which have been used for malignant brain tumors, melanoma, and head-and-neck cancers.^1,6,7^ BPA can accumulate in tumors that overexpress the L-type amino acid transporter 1 (LAT1),^8,9^ but it suffers from limited aqueous solubility and intrinsically low boron payload (one boron atom per molecule, 5.2 wt % of boron). Formulations such as the BPA-fructose complex improve aqueous solubility and enable high-dose administration over a 2-h intravenous infusion (250–500 mg/kg BPA; corresponding to ∼13–25 mg/kg of boron),^10–12^ yet high intracellular boron delivery remains challenging because the boron dose scales directly with the number of BPA molecules delivered. In contrast, BSH provides higher boron content (12 boron atoms per molecule, 59 wt % of boron) and is water-soluble,^13,14^ but it lacks efficient tumor-selective uptake mechanisms, which limit cellular accumulation and therapeutic utility.^15,16^ These limitations motivate boron carrier designs that combine high boron payload with robust aqueous-based formulation and tunable *in vivo* behavior.

Carboranes are boron-rich clusters that offer exceptional boron density together with high chemical and biological stability, making them attractive BNCT building blocks for boron delivery agents.^17^ Their carbon atoms also provide sites for modular derivatization for incorporation into targeted or macromolecular delivery systems. While BSH is a highly water-soluble boron cluster used clinically, its chemistry is more limited for controlled, high-loading conjugation onto carrier scaffolds compared to carboranes.^18,19^ Accordingly, carboranes are often preferred as the boron source for scaffold conjugation. Carborane-based constructs, such as small-molecule carriers,^20,21^ biomolecule conjugates,^22^ and nanocarriers,^23,24^ have been reported; however, carborane hydrophobicity frequently remains a bottleneck, particularly when high payload and aqueous solubility must be achieved simultaneously at a concentration relevant for dosing.^25^ Thus, a key challenge is therefore to improve their aqueous solubility without compromising the effective boron density so that clinically meaningful boron concentrations can be formulated and delivered.

To address these constraints, polymeric platforms provide a direct solution by combining high functional group density, tunable hydrophilic–hydrophobic balance, and controllable architecture through composition and block design.^26,27^ However, most polymeric boron carriers reported to date rely on linear architectures, which can exhibit limited circulation time and rapid clearance depending on size and composition.^28,29^ Star-shaped polymers, made of three or more linear arms extending from a central core, offer an alternative architecture that can increase the local density of functional groups and tune hydrodynamic behavior relative to linear analogues.^30,31^ Importantly, star architectures can support high-density covalent payload incorporation (e.g., drug conjugation) without relying on encapsulation and can be adapted for imaging or theranostic use (combining diagnosis and therapy), allowing biodistribution and pharmacokinetics to be systematically evaluated.^32,33^ For this purpose, poly­(2-oxazoline)­s (POx) are particularly attractive because they are biocompatible and synthetically versatile.^34,35^ POx can be prepared by cationic ring-opening polymerization (CROP), providing well-defined macromolecular structures with narrow dispersity, tunable chain length, and controllable end-group chemistry.^36^ Furthermore, POx allows systematic tuning of hydrophilicity and hydrophobicity through monomer selection, for instance, hydrophilic blocks such as poly­(2-methyl-2-oxazoline) (PMeOx) and poly­(2-ethyl-2-oxazoline) (PEtOx) enhance aqueous solubility and colloidal stability,^37,38^ whereas hydrophobic POx such as poly­(2-butyl-2-oxazoline) (PBuOx) and poly­(2-butyn-2-oxazoline) (PBynOx) introduce hydrophobic segments and reactive functionalities.^39,40^ These features make POx a strong candidate for carborane conjugation strategies aimed at simultaneously improving solubility and maximizing boron payload.

In this work, we developed a modular four-arm star POx-based platform (Figure 1) for carborane delivery and quantitative *in vivo* tracking using positron emission tomography (PET). An ethyl isonipecotate-terminated p­(BynOx-*ran*-MeOx)-*b*-pMeOx (PBMM-EIP) star scaffold bearing alkyne groups on the side chains was functionalized with azidopropyl *meta*-carborane (*m*CB–Pr-N_3_) via copper-catalyzed azide–alkyne cycloaddition (CuAAC) to obtain a star polymer-carborane conjugate (PBMM-*m*CB_n_-EIP; n represents the number of carborane units). We first evaluated *in vitro* cytotoxicity and cellular interaction of different star polymer-carborane conjugates in CAL 27 and FaDu head-and-neck squamous carcinoma cells. To enable real-time pharmacokinetic evaluation *in vivo*, the star end groups were further modified with a 1,4,7,10-tetraazacyclododecane-1,4,7,10-tetraacetic acid (DOTA) chelator and radiolabeled with gallium-68 (*t*_1/2_ = 67.8 min), generating [^68^Ga]­Ga-DOTA-PBMM-*m*CB_20_ for PET imaging and *ex vivo* biodistribution studies in healthy mice. Therefore, the present work was designed as a platform development and pharmacokinetic proof-of-concept study rather than an evaluation of tumor-specific boron delivery or therapeutic BNCT efficacy. Accordingly, tumor boron accumulation, neutron dosimetry, and irradiation efficacy remain to be investigated in future studies.

**Figure 1:**
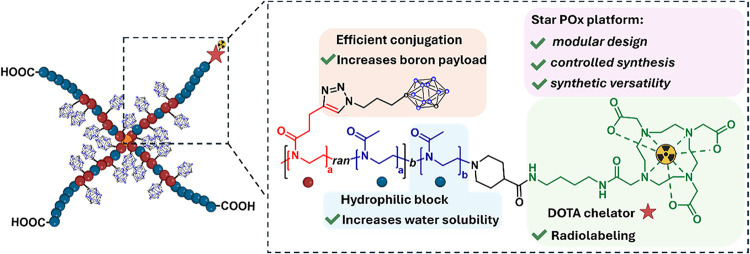
Schematic illustration of the developed four-arm star POx-based scaffold. One representative polymeric arm is magnified to demonstrate its modular architecture, including the boron payload and a DOTA chelator for gallium-68 radiolabeling.

## Experimental Section

### Safety Considerations

Synthesis works with air- and moisture-sensitive agents, and cryogens were carried out using standard Schlenk techniques with appropriate shielding and ventilation; special care must be taken when using liquid N_2_ cold traps to prevent condensation of liquid oxygen. Sodium azide and strong electrophiles/bases (e.g., Tf_2_O and *n*-BuLi) were handled in a dedicated fume hood with appropriate PPE. Cell culture work was conducted in a BSL-2 laboratory. Radioactive materials (e.g., gallium-68) were handled in designated radioisotope laboratories using suitable shielding, contamination control, and personal dosimetry. Animal experiments involving radioactivity were carried out in an authorized facility equipped for small-animal procedures with radionuclides, in accordance with approved institutional animal guidelines and radiation-safety requirements, with appropriate training.

### Materials and Chemicals

All reagents and solvents, including sources, purification, and drying procedures (e.g., solvent distillation and Karl Fischer water content determination), are described in the Supporting Information. Unless otherwise stated, all chemicals and solvents from commercial vendors were used as received.

### Characterizations

Size exclusion chromatography (SEC), nuclear magnetic resonance (NMR), mass spectrometry (MS), dynamic light scattering (DLS), and Fourier transform infrared (FTIR) spectroscopy were used for material characterization throughout the studies. Detailed experimental procedures and complete instrument settings for SEC, NMRs, MS, DLS, and FTIR measurements (including columns, detectors, acquisition parameters, solvents, and operating temperature) are described in the Supporting Information.

### Cationic Ring-Opening Polymerization of Star Polymer (PBMM-EIP)

Pentaerythritol tetrakistriflate (PE­(OTf)_4_)^41^ and 2-butyne-2-oxazoline monomer (BynOx)^42,43^ were synthesized in-house; with full synthetic details provided in the Supporting Information, and characterization data are shown in Figure S1 for PE­(OTf)_4_ and Figure S2 for BynOx. PE­(OTf)_4_ (23.9 mg, 36 μmol, 1 equiv) was dried under vacuum on a Schlenk line for 4 h prior to use, then dissolved in dry ACN (3.5 mL) under N_2_. BynOx (88.7 mg, 720 μmol, 20 equiv) and MeOx (61.3 mg, 720 μmol, 20 equiv) were added sequentially, and the reaction mixture was then heated to 80 °C for 17 h. Monomer conversion was monitored by ^1^H NMR spectroscopy. After completion of the first block, the reaction mixture was cooled to room temperature (RT), and MeOx (735.3 mg, 8.64 mmol, 240 equiv) was added to initiate polymerization of the second block. The reaction mixture was reheated to 80 °C until complete monomer conversion was achieved. The reaction was then cooled to RT and quenched with ethyl isonipecotate (3 equiv. to the triflate groups). The mixture was stirred at 50 °C overnight and subsequently diluted with ∼10 volumes of ACN. The polymer was precipitated into a 10–15-fold excess of ice-cold Et_2_O. The precipitation was repeated twice, and the polymer was freeze-dried to obtain a white powder of PBMM-EIP product (0.8 g, 87% yield). The ethyl ester groups on PBMM-EIP were further deprotected to yield PBMM-COOH for subsequent carborane conjugation. The deprotection protocol is provided in the Supporting Information.

### Synthesis of Star Polymer-Carborane Conjugate (PBMM-*m*CB_20_-EIP) via CuAAC

Prior to the subsequent click reaction, the azidopropyl *meta*-carborane (*m*CB–Pr-N_3_) was prepared by a three-step synthesis; with full synthetic details provided in the Supporting Information, and characterization data are shown in Figure S3. PBMM-*m*CB_20_-EIP, a star polymer-carborane conjugate bearing 20 carborane units, was synthesized via CuAAC as follows. PBMM-EIP (19.6 mg, 1.3 μmol, 1 equiv), *m*CB–Pr-N_3_ (7.1 mg, 31.2 μmol, 24 equiv), PMDETA (10.8 mg, 62.4 μmol, 48 equiv), and DMF (1 mL) were added to a 5 mL flame-dried Schlenk flask. The reaction mixture was degassed by bubbling N_2_ for 20 min. The click reaction was initiated by the addition of CuBr (9 mg, 62.4 μmol, 2 equiv. to azides), and the solution was stirred at RT for 24 h. Then, the solution was concentrated under reduced pressure and purified by SEC using a Sephadex LH-20 column in MeOH. The solvent was removed under a stream of air, and the PBMM-*m*CB_20_-EIP product was lyophilized to obtain as a white powder (18.1 mg, 70% yield).

### Synthesis of DOTA-Modified Star Polymer-Carborane Conjugate (DOTA-PBMM-*m*CB_20_) via Amide Coupling Reaction

Prior to the subsequent coupling step, the terminal end groups of PBMM-*m*CB_20_-EIP were deprotected to yield PBMM-*m*CB_20_-COOH according to the procedure described in the Supporting Information. Amide coupling reaction was carried out as follows. PBMM-*m*CB_20_-COOH (8.1 mg, 0.62 μmol, 1 equiv) was dissolved in dry DMF (0.2 mL) in a flame-dried Schlenk flask. Then, HATU (1.22 mg, 3.22 μmol, 5.2 equiv) and DIPEA (0.83 mg, 6.45 μmol, 10.4 equiv) were added at RT. The reaction mixture was cooled in an ice bath for 30 min and then allowed to warm to RT. Subsequently, 4-aminobutyl-DOTA-tris­(*t*-butyl ester) (7.82 mg, 9.92 μmol, 16 equiv) was added, and the reaction was stirred at RT for 24 h. The solvent was removed under reduced pressure. The crude product was purified by SEC on a Sephadex LH-20 column using MeOH as the eluent. The solvent was removed under a stream of air, and the DOTA-PBMM-*m*CB_20_ was lyophilized to obtain a white powder (8.0 mg, 80% yield).

### Aqueous Solubility of Star Polymer-Carborane Conjugate

The aqueous solubility of PBMM-*m*CB_20_-EIP was assessed by stepwise water titration with visual observation. Briefly, PBMM-*m*CB_20_-EIP (10 mg) was weighed into a 2 mL glass vial, and dH_2_O was added in small increments until complete dissolution was observed (*n* = 4). An initial aliquot of dH_2_O (25 μL) was added in an attempt to dissolve the material; however, the material did not completely dissolve. Additional dH_2_O (8.3 μL) was then added (total dH_2_O volume 33.3 μL), and the sample was sonicated for 30 min. During sonication, the vial was inspected every 10 min until the solution became visually clear with no undissolved material. Because polymer volume is non-negligible at this high concentration, solubility was not calculated solely from the volume of water added. Instead, an apparent solubility (g/L) was calculated by dividing the polymer mass by the final solution volume measured after complete dissolution using a Hamilton microsyringe (45 ± 1 μL; ± 1% accuracy of nominal volume). Moreover, the concentration is also reported as a mass fraction (w/w), calculated from polymer mass relative to the total mass of polymer and dH_2_O.

### Cell Culture

The cell lines used in this study were the human squamous cell carcinoma (HNSCC) cell lines CAL 27 (CRL-2095) and FaDu (HTB-43), obtained from the American Type Culture Collection (ATCC, Manassas, VA). General cell culture materials and conditions for *in vitro* and *in vivo* studies are provided in the Supporting Information.

### *In Vitro* Cell Cytotoxicity

CAL 27 and FaDu cells were seeded in white-walled 96-well plates (5000 cells/well, viability >97%) in 100 μL of the corresponding complete medium and allowed to attach overnight. The medium was then aspirated and replaced with 100 μL of fresh medium containing PBMM-*m*CB_10_-EIP, PBMM-*m*CB_20_-EIP, or BSH. For each treatment group, the total boron concentration in the incubation medium was kept constant across formulations (5, 10, 50, 125, and 250 μM boron concentration). An unconjugated PBMM-EIP matched to the corresponding boron concentrations was included as the vehicle control. At these fixed boron concentrations, the corresponding PBMM-EIP concentrations were 0.72, 14.4, 72, 180, and 360 μg/mL, respectively. Untreated cells (complete medium only) and 1% (v/v) Triton X-100 in culture medium served as negative and positive controls, respectively. Cells were incubated for 6 and 24 h at 37 °C and 5% CO_2_. At each time point, the medium was removed, and cells were washed twice with 100 μL of prewarmed 1× DPBS (pH 7.4). CellTiter-Glo reagent diluted 1:1 (v/v) with 1× DPBS was then added (50 μL/well), and plates were briefly mixed, protected from light, and incubated at RT for 15 min to allow cell lysis and luminescence signal stabilization. Luminescence was measured using a Synergy H1 Hybrid multimode microplate reader (BioTek, Winooski, VT). All conditions were tested in quadruplicate (*n* = 4).

### Cellular Interaction

CAL 27 and FaDu cells were seeded in transparent 6-well plates (1 × 10^6^ cells/well, viability >98%) in 1 mL of the corresponding complete medium and allowed to attach overnight. The medium was then replaced with 1 mL of fresh medium containing PBMM-*m*CB_20_-EIP or BSH at boron-equivalent concentrations of 100 μM, and cells were further incubated for 24 h. After incubation, the treatment medium was discarded, and cells were washed twice with 0.5 mL of prewarmed 1× DPBS (pH 7.4). Cells were detached by adding 0.5 mL of TrypLE Express per well and incubating at 37 °C for 7 min. Cell suspensions from six wells per condition were pooled into a 15 mL conical tube and neutralized with complete culture medium. Cells were pelleted by centrifugation (130*g*, 5 min, 4 °C), the supernatant was aspirated, and the pellet was resuspended in 5 mL of complete medium. Total cell number and viability were determined using an automated cell counter. Cell-associated boron concentration was quantified by microwave plasma atomic emission spectroscopy (MP-AES) following nitric acid digestion of collected cell samples. Complete details on acid digestion, sample preparation, instrument parameters, and data normalization procedures are provided in the Supporting Information.

### Radiolabeling

Radiolabeling solutions were prepared in ultrapure water rendered metal-free by pretreatment with Chelex 100 ion-exchange resin (Sigma-Aldrich, Saint Louis, MO). [^68^Ga]­GaCl_3_ was eluted from a GalliaPharm ^68^Ge/^68^Ga generator (Eckert & Ziegler Medical, Berlin, Germany) using 10 mL of ultrapure 0.1 M HCl. The eluate was passed through a strong cationic-exchange (SCX) silica cartridge (Eichrom Technologies LLC, Lisle, IL) to trap gallium-68, which was then eluted and concentrated in 250 μL of acidified NaCl solution (5.5 M HCl/5 M NaCl, 1:40 v/v). DOTA-PBMM-*m*CB_20_ (250 μL, 5 mg/mL) was dispersed in 0.25 M ammonium acetate buffer (700 μL, pH 5.5) in a Protein LoBind tube (Eppendorf, Hamburg, Germany). Ascorbic acid (100 μL, 20 mg/mL) and concentrated [^68^Ga]­GaCl_3_ (100 μL) were then added. The final reaction mixture contained DOTA-PBMM-*m*CB_20_ (1.1 mg/mL) in ammonium acetate buffer (0.15 M, pH 4.5) and ascorbic acid (1.74 mg/mL). Radiolabeling was carried out at 95 °C for 30 min. The reaction was quenched by the addition of 50 mM diethylenetriaminepentaacetic acid (DTPA) solution (2 μL) and allowed to cool down to RT. The crude reaction mixture was loaded onto an Amicon centrifugal filter unit (MWCO 10 kDa) and centrifuged at 7500*g* for 5 min. The retentate containing [^68^Ga]­Ga-DOTA-PBMM-*m*CB_20_ was further washed once with 20 mM sodium citrate (500 μL) to remove nonchelated and nonspecifically bound gallium-68, followed by solvent exchange into 0.9% isotonic saline over two centrifugation cycles. Quality control was carried out by radio instant thin-layer chromatography (radio-iTLC); full details are provided in the Supporting Information.

### Radiolabel Stability

The radiolabel stability of [^68^Ga]­Ga-DOTA-PBMM-*m*CB_20_ was evaluated under physiologically relevant conditions, including 0.9% isotonic saline, 50% human plasma in 1× PBS (pH 7.4), and 0.2 mM FeCl_3_ solution. Purified [^68^Ga]­Ga-DOTA-PBMM-*m*CB_20_ (50 μg, ∼1.4 MBq) was dispersed in 0.5 mL of each medium and incubated at 37 °C with constant shaking at 350 rpm. At predetermined time points (15, 30, 60, 120, and 180 min), aliquots (5 μL) were drawn and analyzed by radio-iTLC to quantify the fraction of intact radiolabeled conjugate, using the procedure described in the quality control of radiolabeling. All experiments were carried out in triplicate (*n* = 3).

### PET/CT Imaging and *Ex Vivo* Biodistribution

All animal experiments were conducted following the protocols evaluated and approved by the National Board of Animal Experimentation in Finland (ethics approval number: ESAVI/17892/2025) and in accordance with European Union legislation (Directive 2010/63/EU). Full details of the animal model, housing, and husbandry are provided in the Supporting Information.

Whole-body PET/CT was conducted as a 90 min dynamic acquisition (frames: 6 × 10 s, 4 × 1 min, 5 × 5 min, 6 × 10 min) following intravenous administration of [^68^Ga]­Ga-DOTA-PBMM-*m*CB_20_ (3.5 ± 0.1 MBq, 200 μg in 100 μL of 0.9% isotonic saline). Mice (*n* = 3) were anesthetized with 2.5–3% isoflurane in medical oxygen (1 L/min) and imaged using a Molecubes benchtop β-CUBE for PET and X-CUBE for micro-CT (Molecubes NV, Gent, Belgium). For *ex vivo* biodistribution, animals (*n* = 4) were euthanized at 90 min postinjection (p.i.) or immediately after PET/CT imaging. Selected organs were collected for radioactivity counting using an automatic γ counter 1480 Wizard 3″ (PerkinElmer Life Sciences, Waltham, MA). Image analysis was carried out using VivoQuant 2021 (InviCRO LLC, Needham, MA). PET data are reported as standardized uptake values (SUV), a unitless metric calculated as tissue activity concentration divided by injected activity normalized to the body weight of the animal, and *ex vivo* biodistribution from γ counting is reported as percent injected dose per gram of tissue (%ID/g).

### Statistical Analysis

Statistical analysis and graphical plots were done using OriginPro 2026. Quantitative data are presented as mean ± standard deviation (SD), and the number (*n*) of replicates or animal numbers is reported where appropriate. Direct comparisons between sample groups in cytotoxicity studies were carried out using unpaired Student’s *t*-tests, with statistical significance defined as *p < 0.05, **p < 0.01, and ***p < 0.001.

## Results and Discussion

### Synthesis and Characterization of Star Polymer

To generate the star polymer-carborane conjugate, first, a four-arm star block copolymer p­(BynOx-*ran*-MeOx)-*b*-pMeOx bearing alkyne-functionalized side chains and ethyl isonipecotate end groups (PBMM-EIP) was designed and synthesized using PE­(OTf)_4_ as a tetrameric initiator. The first inner block comprised a copolymer of hydrophilic MeOx and hydrophobic BynOx. MeOx was introduced as an internal diluent to reduce potential steric hindrance around the alkyne-bearing side chains and thereby facilitate efficient covalent coupling to bulky *m*CB–Pr-N_3_. Subsequently, a second PMeOx block was polymerized to improve the overall aqueous solubility of the star polymers. To introduce reactive end-groups, polymerization was terminated with ethyl isonipecotate, a derivative of piperidine. The resulting terminal ethyl esters were then deprotected to yield PBMM-COOH with free carboxylic acid groups available for subsequent conjugation (Figure 2).^44^

**Figure 2:**
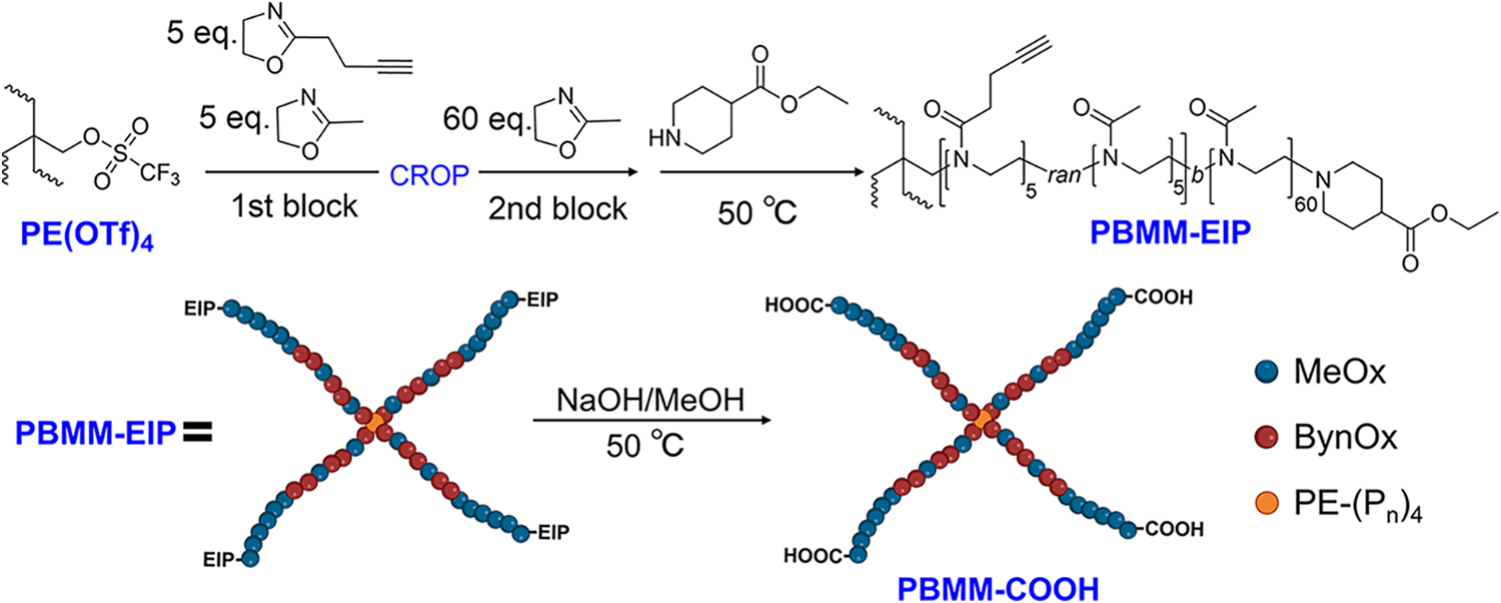
Overview of the synthetic route of the star polymer. Upper panel: synthesis of PBMM-EIP via CROP of BynOx and MeOx using PE­(OTf)_4_ as the initiator and ethyl isonipecotate (EIP) as the terminating agent. Lower panel: alkaline deprotection of EIP to yield the carboxyl-terminated star polymer (PBMM-COOH).

To confirm the successful synthesis of PBMM-EIP, ^1^H NMR and SEC were conducted, with key results summarized in Table S1. The ^1^H NMR spectrum showed all signals expected for the targeted star polymer structure, including signals at δ 3.1–3.7 ppm (polymer backbone), 2.7–2.4 ppm (−CH_2_CH_2_– of BynOx), and 2.1–2.0 ppm (−CH_3_ of MeOx), indicating the successful formation of PBMM-EIP (Figure S4A,B). SEC analysis revealed a single, symmetric peak with a low dispersity (*Đ* = 1.15), indicating a well-defined PBMM-EIP with narrow molar-mass distribution (Figure S4C). The number-average molar mass was derived from the SEC elution profile. As commonly observed for star polymers, PBMM-EIP exhibited lower apparent molar masses (*M*_n, SEC_) than the corresponding theoretical molecular weight (*M*_n, theo_), which attributes to the compact star-shaped architecture and its reduced hydrodynamic volume relative to linear analogues.^30^ Unfortunately, *M*_n_ could not be determined by ^1^H NMR because the PE­(OTf)_4_ initiator signals overlapped with the polymer backbone resonances, and termination with EIP was incomplete, which prevented reliable end-group integration.

### Synthesis and Characterization of Star Polymer-Carborane Conjugate

For the synthesis of star polymer-carborane conjugates (PBMM-*m*CB_n_-EIP), *m*CB–Pr-N_3_ was utilized as a boron cluster building block. The conjugation reaction between the alkyne-functionalized star polymer PBMM-EIP and azide-modified boron cluster was conducted via a CuAAC reaction, as shown in Figure 3.

**Figure 3:**
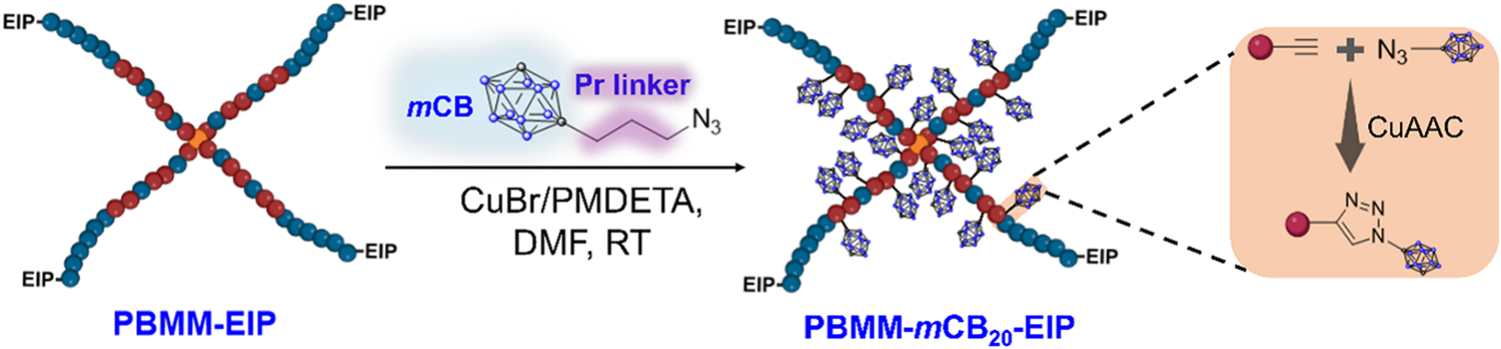
Schematic illustration of the synthetic route to the star polymer-carborane conjugate. Conjugation of azidopropyl *meta*-carborane (*m*CB–Pr-N_3_) to PBMM-EIP via CuAAC reaction to yield star polymer-carborane conjugate (PBMM-*m*CB_20_-EIP).

When 12 equiv of *m*CB–Pr-N_3_ (0.6 equiv. to alkynes) was used, ^1^H NMR analysis indicated attachment of an average of ∼10 carboranes per star polymer, herein referred to as PBMM-*m*CB_10_-EIP. The complete synthetic protocol and characterization of PBMM-*m*CB_10_-EIP are provided in the Supporting Information and Figure S5, respectively. To maximize the amount of boron payloads, 24 equiv of *m*CB–Pr-N_3_ (1.2 equiv. to alkynes) was used to drive conjugation of all alkyne groups on the PBMM-EIP arms. Because the synthesized PBMM-EIP contains 20 alkyne groups in total, this approach yielded PBMM-*m*CB_20_-EIP (Figure 4A) bearing 20 carborane units. The success of this reaction was assessed by conducting a comprehensive characterization of PBMM-*m*CB_20_-EIP using ^1^H-, ^1^H DOSY-NMRs, and FTIR. The ^1^H NMR integrals shown in Figure 4B were normalized to a single arm of the star polymer. Comparison of the methyl resonance (H^6^) and the triazole signal (H^9^) indicated approximately five carboranes per arm, suggesting complete conversion of the alkyne groups. ^1^H DOSY NMR spectrum (Figure 4C) of purified PBMM-*m*CB_20_-EIP showed a single diffusing species, thereby confirming the covalent attachment of *m*CB–Pr-N_3_ to the polymer backbone and effective removal of unbound components. Moreover, in the FTIR spectra (Figure 4D), the azide stretching observed in the carborane conjugation block at ∼2100 cm^–1^ was no longer observed after the reaction, while bands associated with B–H stretching ∼2600 cm^–1^ remained in the purified conjugate, consistent with the expectations of a successful coupling reaction between the carboranes and the star polymer. Additional ^11^B NMR and SEC characterization of PBMM-*m*CB_20_-EIP are provided in Figure S6 and Table S1, respectively. Altogether, these results confirmed that a well-defined star polymer-carborane conjugate had been successfully constructed.

**Figure 4:**
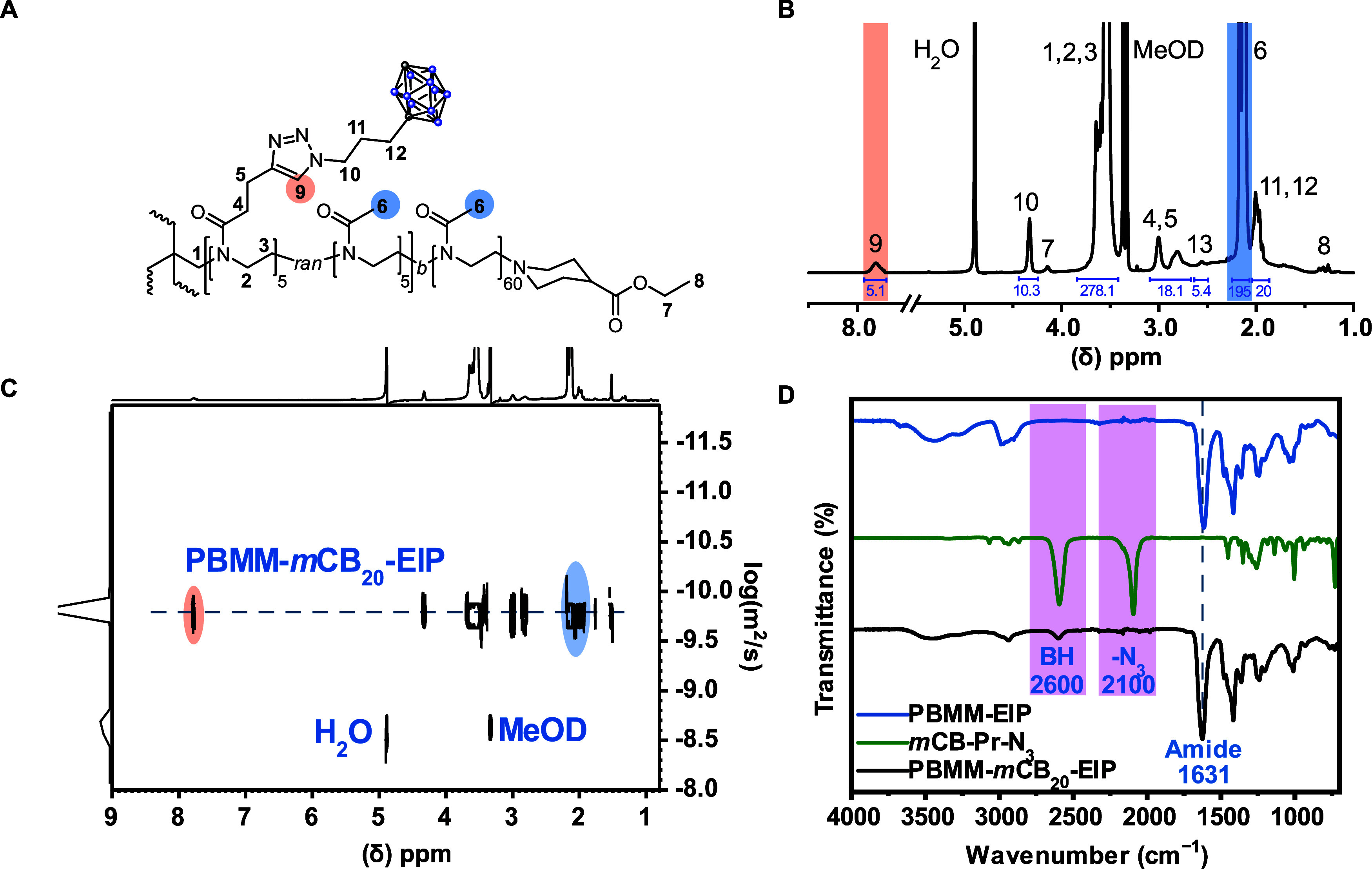
PBMM-*m*CB_20_-EIP characterization. (A) Chemical structure of PBMM-*m*CB_20_-EIP, (B) expanded region of the ^1^H NMR spectrum of PBMM-*m*CB_20_-EIP in MeOD, (C) ^1^H DOSY NMR spectrum of PBMM-*m*CB_20_-EIP in MeOD, (D) overlaid FTIR spectra of PBMM-EIP, *m*CB–Pr-N_3_ starting material, and the PBMM-*m*CB_20_-EIP conjugate.

### Synthesis and Characterization of DOTA-Modified Star Polymer-Carborane Conjugate

To enable real-time PET imaging and *ex vivo* biodistribution, the deprotected star polymer PBMM-COOH was used to prepare PBMM-*m*CB_20_-COOH. The complete characterization (e.g., ^1^H NMR and SEC chromatograms) of PBMM-COOH is provided in Figure S7. Subsequent conjugation of a DOTA chelator and PBMM-*m*CB_20_-COOH by an amide coupling reaction yielded the DOTA-PBMM-*m*CB_20_ ready for gallium-68 radiolabeling. The ^1^H and ^1^H DOSY NMR spectra of PBMM-*m*CB_20_-COOH and DOTA-PBMM-*m*CB_20_ are provided in Figures S8 and S9, respectively.

### Size Distribution of Star Polymer and Star Polymer-Carborane Conjugate

DLS measurements were conducted to evaluate the size and size distribution of PBMM-EIP and PBMM-*m*CB_20_-EIP. The experimental intensity autocorrelation functions showed a good agreement with the fitted curves (Figure S10). The hydrodynamic diameter (*D*_h_) of both samples was initially measured at a concentration of 1 g/L at 25 °C. At this concentration, only PBMM-*m*CB_20_-EIP provided sufficient scattering intensity (353 kcps) for robust analysis, whereas PBMM-EIP produced an insufficient signal (14 kcps). The PBMM-EIP concentration was therefore increased to 10 g/L, resulting in adequate and reliable count rates (120 kcps). Nevertheless, it should be noted that both measurements were conducted under dilute conditions, where interparticle interactions are minimal, and the diffusion coefficient approaches the infinite-dilution limit; consequently, the derived *D*_h_ is not expected to be concentration-dependent.^45^

The intensity-weighted DLS size distributions (Figure 5A,5D) revealed two populations of PBMM-EIP, with *D*_h_ of ∼8 and 123 nm, which were assigned to individual stars (unimers) and a minor fraction of aggregates, respectively. After conjugation with *m*CB–Pr-N_3_, the dominant *D*_h_ shifted to a larger size, with *D*_h_ increasing to ∼28 nm for PBMM-*m*CB_20_-EIP, consistent with an increased hydrodynamic volume that may arise from carborane-induced changes in chain conformation and/or intermolecular association. To estimate which species predominated in the solution by their number, the intensity distributions were further converted to volume- and number-weighted distributions assuming that they are hard spheres. In the volume-weighted distributions (Figure 5E), the smaller size population remained dominant for both PBMM-EIP and PBMM-*m*CB_20_-EIP, while the contribution of the larger species was markedly decreased. In the number-weighted distributions (Figure 5C,5F), only the smaller population was evident, and the larger species became negligible. These results are consistent with PBMM-EIP and PBMM-*m*CB_20_-EIP predominantly existing as their smaller populations in aqueous solution, with only a minor fraction of larger assemblies present.

**Figure 5:**
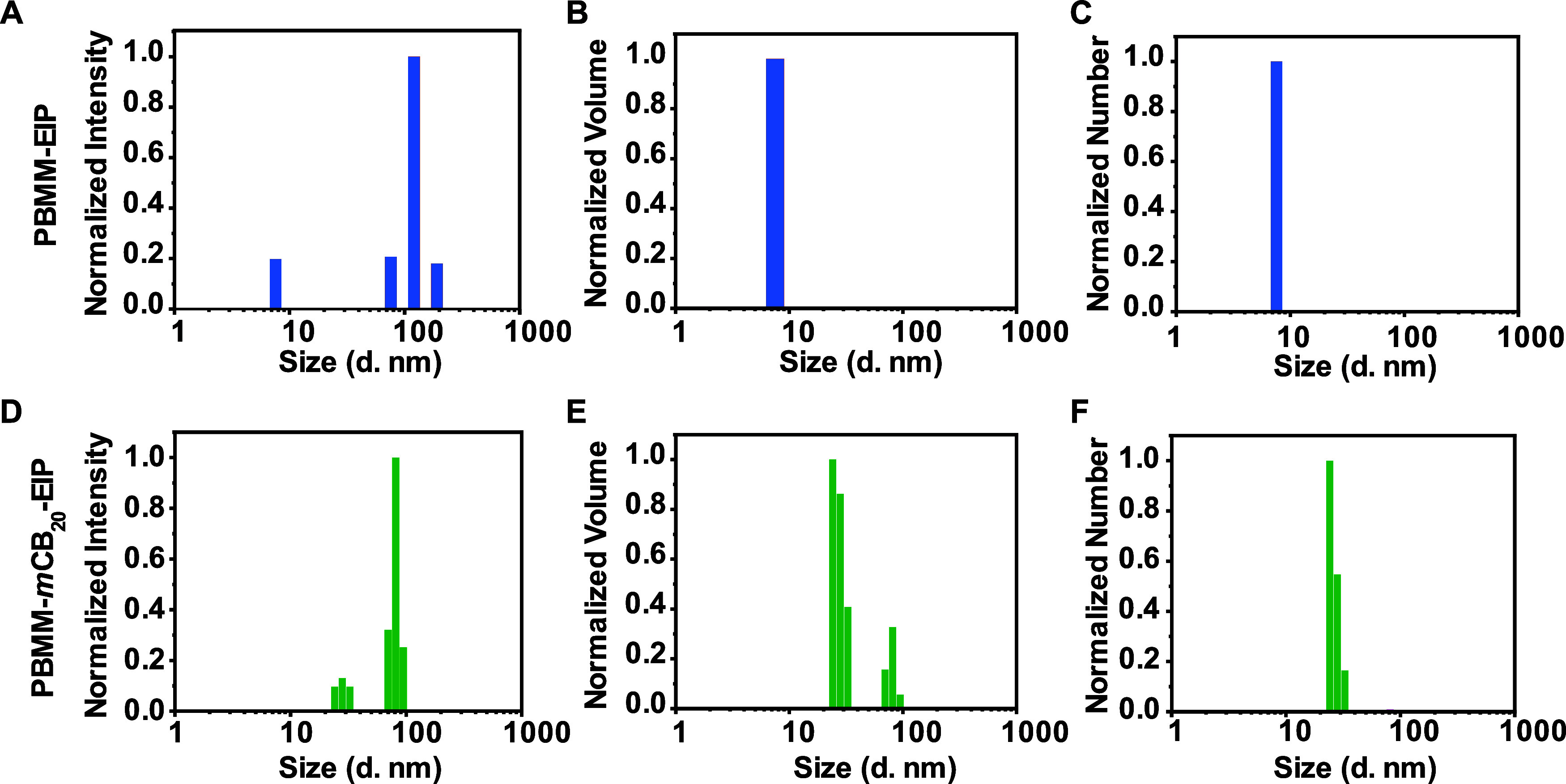
DLS size distributions normalized to intensity, volume, and number for PBMM-EIP (A–C) at 10 g/L and PBMM-*m*CB_20_-EIP (D–F) at 1 g/L. Measurements were carried out with 0.45-μm filtered samples at a scattering angle of 90°.

### Aqueous Solubility of Star Polymer-Carborane Conjugate

Given the context of boron carrier formulation, aqueous solubility is a critical parameter for carborane-containing carriers, as it defines the maximum boron concentration that can be formulated for intravenous administration. We therefore quantified the boron amount that could be solubilized in aqueous medium using the fully loaded conjugate PBMM-*m*CB_20_-EIP. Since dissolution of the unmodified star polymer (PBMM-EIP) was readily achieved, solubility of PBMM-*m*CB_20_-EIP was assessed by direct visual observation during stepwise addition of water to the solid polymer (Figure S11); no solvent switch or similar was required. PBMM-*m*CB_20_-EIP dissolved completely at a concentration of 225 ± 5 g/L (196 ± 22 mg/g), corresponding to an estimated boron concentration of 26 ± 1 g/L of boron. In comparison, the clinically used BPA-fructose complex is typically formulated at 30 g/L (∼1.5 g/L of boron),^46^ indicating that PBMM-*m*CB_20_-EIP can achieve substantially higher boron concentration in aqueous formulation (∼17 times higher on a boron basis) without the need for any additional solubilizer. It should also be noted that since POx are highly soluble due to the tertiary amides in each repeating unit, their solubility does not depend much on charges, and therefore pH. On this basis, the formulation of PBMM-*m*CB_20_-EIP is expected to accommodate boron dose in a comparable range, and potentially higher, while offering flexibility in infusion concentration and volume without relying on solubilizing excipients.

### *In Vitro* Cytotoxicity

While POx in general, including nonionic amphiphilic POx, typically exhibit low cytotoxicity,^47^ amphiphilic star-POx conjugates have rarely been investigated. Therefore, we evaluated the *in vitro* cytocompatibility as an initial screen for assessing the cellular toxicity of the developed star polymer-carborane conjugates. Two HNSCC cell lines (CAL 27 and FaDu) were selected as clinically relevant models for BNCT studies of tongue and hypopharyngeal squamous cell carcinoma, respectively. Cells were incubated with PBMM-*m*CB_10_-EIP and PBMM-*m*CB_20_-EIP at boron concentrations of 5–250 μM for 6 and 24 h, with BSH and PBMM-EIP included as a clinical reference and the empty carrier control at equivalent concentrations, respectively. The tested concentration range and exposure times reflected practical formulation levels and estimated contact times following intravenous administration. Across both cell lines, PBMM-EIP, PBMM-*m*CB_10_-EIP, and PBMM-*m*CB_20_-EIP maintained high viability, generally remaining above 80% at all concentrations and at both time points (Figure 6). In contrast, BSH-treated cells showed a modest trend toward lower viability at higher concentrations (>125 μM) at 24 h in the CAL 27 cells and at both time points in the FaDu cells. While the underlying mechanism was not investigated here, this difference may reflect the distinct molecular properties of BSH relative to the macromolecular star polymer carriers. Overall, these results suggest that star polymer-carborane conjugates are well tolerated *in vitro* in the investigated concentration range and exposure times, indicating that carborane modification did not introduce acute toxicity in these HNSCC cell models. From a translational perspective, low cytotoxicity is an important requirement for BNCT carriers, which often need to be administered at relatively high doses to achieve therapeutic intratumoral boron concentrations. Therefore, maintaining cell viability helps ensure that subsequent uptake and retention reflect cellular interaction rather than toxicity-driven changes in metabolism or disruption of membrane integrity.

**Figure 6:**
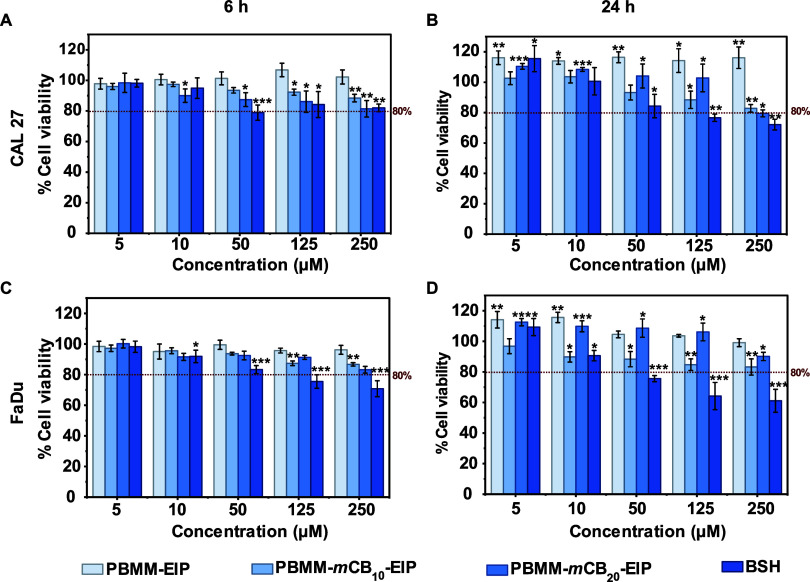
*In vitro* cytotoxicity studies in HNSCC (A, B) CAL 27 and (C, D) FaDu cell lines after incubation with PBMM-EIP, PBMM-*m*CB_10_-EIP, PBMM-*m*CB_20_-EIP, and clinically relevant BSH at boron concentrations of 5, 10, 50, 125, and 250 μM (corresponding to polymer concentrations of 0.72, 14.4, 72, 180, and 360 μg/mL) for 6 and 24 h. Negative and positive controls were complete cell culture medium and 1% (v/v) Triton X-100 solution, respectively (data not shown). Data are shown as mean ± SD (*n* = 4). Statistical significance (*p*-value) was assessed using unpaired Student’s *t*-tests in comparison with the negative control, where the viability was set to 100% (*p < 0.05, **p < 0.01, and ***p < 0.001). The dashed line indicates the 80% viability threshold.

### Cellular Interaction

For BNCT to be effective, it is beneficial if the boron is localized within or in close proximity to tumor cells. We further conducted cellular interaction studies in both CAL 27 and FaDu cells. Since both PBMM-*m*CB_10_-EIP and PBMM-*m*CB_20_-EIP generally exhibited good cytocompatibility, PBMM-*m*CB_20_-EIP was only advanced as the lead construct due to its higher boron payload. BSH was also included as a reference compound. PBMM-*m*CB_20_-EIP and BSH were prepared to provide a nominal boron concentration of 100 μM. Cellular interaction was evaluated after 24 h exposure to monitor not only initial association but also longer-term exposure under sustained contact. Under these conditions, PBMM-*m*CB_20_-EIP revealed a higher cell-associated boron concentration than BSH in both CAL 27 and FaDu cells, quantified by MP-AES after washing and expressed as an absolute boron content (ng) per 1 × 10^6^ cells (Figure 7). This outcome is consistent with the distinct physicochemical nature of the two agents. BSH is a small and highly hydrophilic boron cluster that typically has limited passive membrane permeability and can be prone to washout, whereas PBMM-*m*CB_20_-EIP carries multiple hydrophobic carboranes on a macromolecular scaffold, which likely dominates the endocytosis behavior. As a result, the star polymer-carborane conjugate may exhibit stronger membrane association, greater cellular retention, or uptake through endocytic pathways, which could contribute to the higher cell-associated boron concentration after extended incubation.^48,49^ However, the present method quantified total cell-associated boron and did not distinguish internalized boron from boron remaining associated with the cell surface. Therefore, the underlying mechanism for cellular association cannot be determined from the present data.

**Figure 7:**
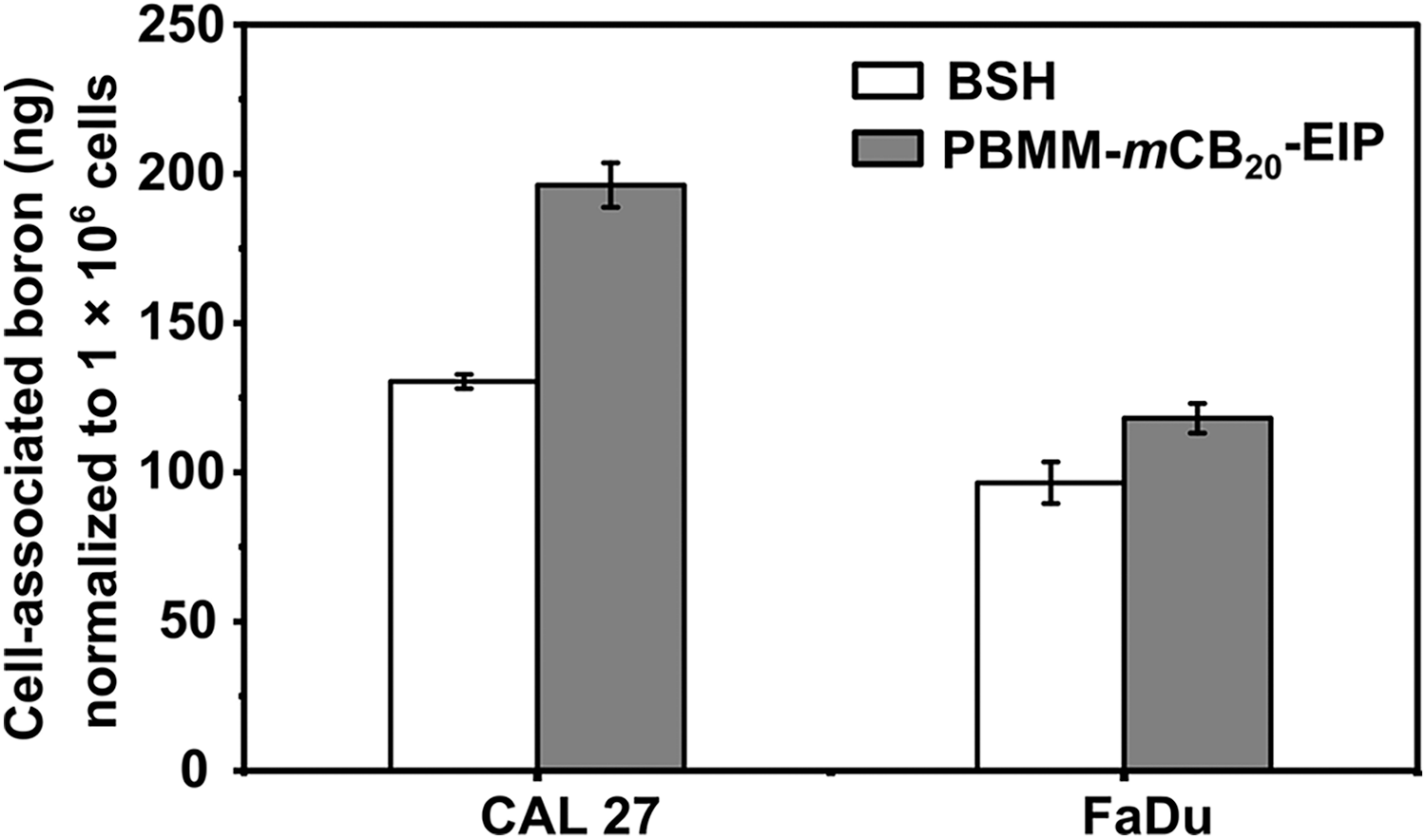
Cellular interaction after 24 h incubation in HNSCC cell lines (CAL 27 and FaDu), quantified by MP-AES as cell-associated boron (ng) normalized to 1 × 10^6^ cells following incubation with PBMM-*m*CB_20_-EIP (nominal 100 μM boron, based on ^1^H NMR) or BSH (100 μM) (*n* = 3).

Importantly, observing the same trend in two HNSCC cell lines suggests that the physicochemical properties of the star polymer-carborane conjugate likely contribute to its higher cellular interaction, although the underlying mechanisms were not investigated in the present study. This comparison should therefore be interpreted only in relation to BSH and should not be extrapolated to other boron delivery agents. In particular, clinically used BPA represents a mechanistically distinct benchmark, because its uptake is mediated by amino acid transporters, especially LAT1. In contrast, the cellular and tumor accumulation of nontargeted macromolecular carriers depends on various factors, such as vascular permeability, interstitial transport, membrane interactions, circulation, and clearance. To further enhance tumor and cellular targeting, future work could utilize macromolecular engineering or incorporate targeting ligands at the star-polymer termini. Optimization of ligand identity and density, followed by direct comparison with BPA in tumor-bearing models, will be required to determine whether these modifications improve the tumor boron concentration and tumor-to-background ratios.

### Radiolabeling and Radiolabel Stability

To enable real-time monitoring of *in vivo* pharmacokinetic behavior of the star polymer-carborane conjugate using PET imaging, a gallium-68-labeled DOTA-PBMM-*m*CB_20_ analogue was prepared. DOTA-conjugated PBMM-*m*CB_20_ was synthesized and radiolabeled with [^68^Ga]­GaCl_3_ to generate [^68^Ga]­Ga-DOTA-PBMM-*m*CB_20_. Overall, radiolabeling proceeded with good radiochemical conversion (RCC = 84 ± 10%, *n* = 3), yielding a radiolabeled star polymer-carborane conjugate with modest radiochemical yield (RCY = 37 ± 11%, *n* = 3) but high radiochemical purity (RCP = 98 ± 1%, *n* = 3) after purification. The apparent specific activity of [^68^Ga]­Ga-DOTA-PBMM-*m*CB_20_ was 27 ± 6 MBq/mg (*n* = 3), which was sufficient for subsequent animal studies.

Radiolabel stability was assessed *in vitro* prior to animal dosing to support that the PET signal reflects stable [^68^Ga]­Ga-DOTA-PBMM-*m*CB_20_ rather than released or transchelated gallium-68 species. The radiotracer was incubated at 37 °C in sterile 0.9% isotonic saline (injection formulation), 50% human plasma, and 0.2 mM FeCl_3_ as a stringent metal-challenge condition. The FeCl_3_ concentration represents a large excess relative to circulating iron (0.01–0.03 mM) in the human blood and was included to monitor potential transmetalation and competitive metal binding that could compromise the stability of [^68^Ga]­Ga-DOTA complex. In parallel, plasma incubation was used to evaluate the influence of blood components and protein-associated interactions expected in systemic circulation. Under all tested conditions, radio-iTLC analysis demonstrated that radiolabeled complex remained above 98% intact over 180 min (Figure S12), indicating high thermodynamic stability of the radiolabeled complex at least within the planned PET imaging window and confirming the suitability of the formulation for intravenous injection.

### PET/CT Imaging and *Ex Vivo* Biodistribution

Whole-body PET/CT images of [^68^Ga]­Ga-DOTA-PBMM-*m*CB_20_ (200 μg, ∼3.5 MBq) at 90 min p.i. (Figure 8A) revealed prominent radioactivity in the urinary bladder, with additional activity in the kidneys and the heart, consistent with rapid clearance through the renal pathway and a persistent blood-pool signal for [^68^Ga]­Ga-DOTA-PBMM-*m*CB_20_ within the gallium-68 PET imaging time frame. Based on the injected formulation, the administered carborane dose was 1.7 mg/kg body weight, corresponding to ∼1.3 mg/kg of boron (estimated from the boron mass of the *m*CB units). This tracer-level dose was chosen to characterize the distribution and clearance of the developed carrier *in vivo*, rather than to mimic therapeutic BNCT dosing regimens that typically use controlled long infusion time to maintain boron concentration in plasma. Accordingly, the present PET study provides a pharmacokinetic baseline for the carrier. It should be noted that conversion of PET-derived SUV values (uptake normalized to injected dose and body weight) into absolute boron concentrations (boron mass per tissue mass) will require correlated PET analysis and direct tissue boron quantification in future dose escalation studies in a tumor model.

**Figure 8:**
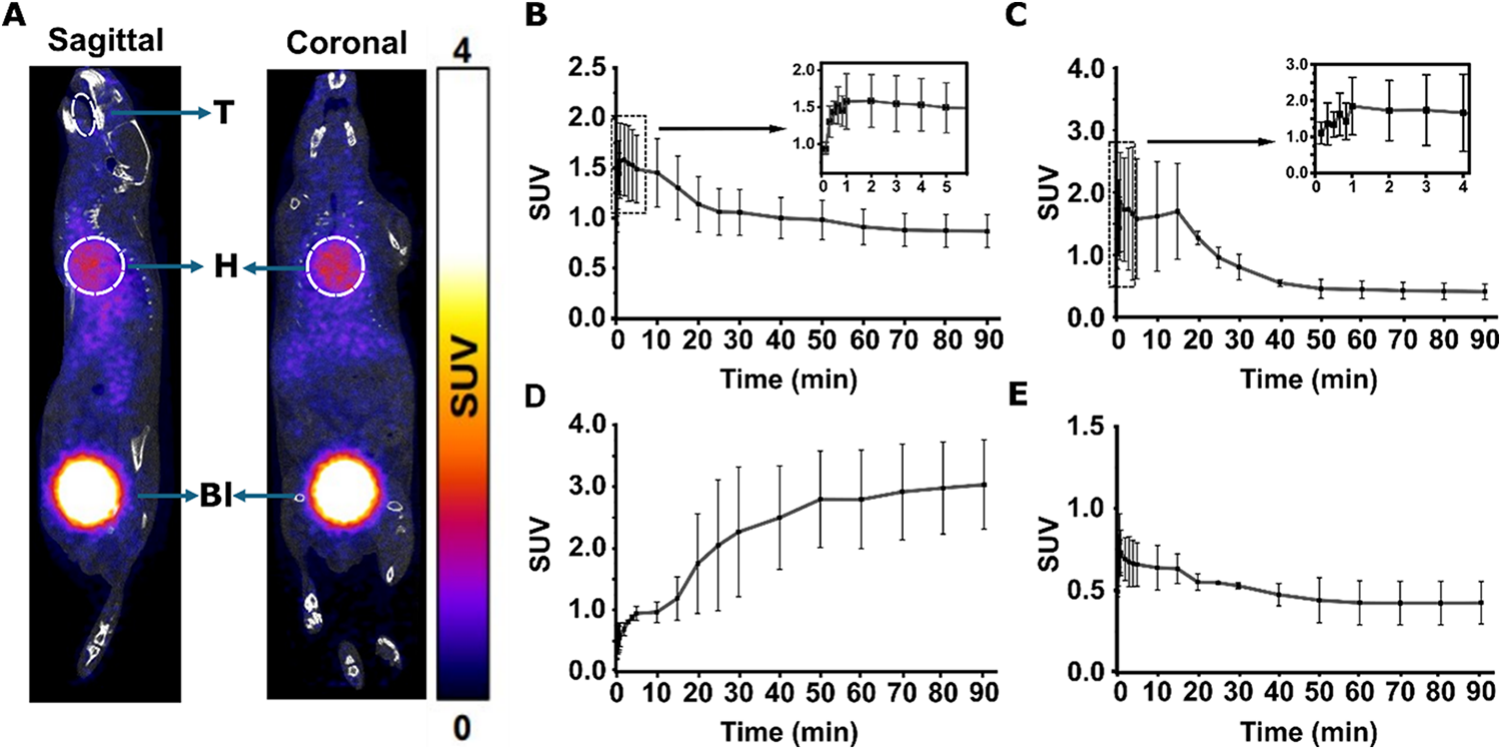
(A) Representative PET/CT images in sagittal and coronal planes at 90 min following intravenous injection of [^68^Ga]­Ga-DOTA-PBMM-*m*CB_20_ (200 μg, ∼3.5 MBq) in healthy mice, showing a high blood-pool activity in the heart (H) and predominant renal clearance through bladder (Bl) with negligible physiological uptake in the healthy tongue (T). Time-activity curves expressed as standardized uptake value (SUV) for the (B) heart, (C) kidneys, (D) bladder, and (E) liver (*n* = 3). Insets in B and C show magnified views of the early distribution phase at 0–5 min for the heart and 0–4 min for the kidneys, respectively.

In the present study, dynamic PET acquisition allowed time-activity-curve (TAC) analysis generated from regions of interest (ROIs) drawn over the heart (blood pool), kidneys, bladder, and liver. Heart activity (Figure 8B) represented as SUV_mean_ exhibited an initial peak of 1.58 ± 0.36 at 2 min, followed by a gradual decline to 0.87 ± 0.16 at 90 min, corresponding to an area-under-curve value (AUC_0–90 min_) of 95 SUV·min, which represents the cumulative blood-pool activity over the scan. In parallel, kidney activity (Figure 8C) also peaked early at 3 min (SUV_mean_ = 1.73 ± 0.98), then declined and approached a plateau at 50 min (SUV_mean_ = 0.43 ± 0.14), yielding an AUC_0–90 min_ of 71 SUV·min. In contrast, bladder activity (Figure 8D) increased continuously throughout the scan, reaching SUV_mean_ of 3.03 ± 0.72 (AUC_0–90 min_ = 208 SUV·min) at 90 min, indicating cumulative urinary excretion. This temporal pattern of early renal activity followed by progressive bladder accumulation suggests the characteristic of predominant renal clearance of [^68^Ga]­Ga-DOTA-PBMM-*m*CB_20_, a profile often observed for hydrophilic POx-based constructs. To illustrate [^68^Ga]­Ga-DOTA-PBMM-*m*CB_20_ pharmacokinetics in more detail, the dynamic data set was further presented as representative PET images over three intervals (0–15 min, 15–60 min, and 60–90 min; Figure S13 and Supporting Video S1). These PET frames show rapid early activity distribution in the kidneys and blood pool, followed by progressive accumulation in the bladder with declining activity in the blood pool and kidneys. Across all time windows, hepatic activity remained constant over the scan duration.

*Ex vivo* biodistribution at 90 min p.i. (Figure S14) corroborated the PET imaging findings, with remarkably high activity detected in urine (557 ± 290%ID/g), reflecting rapid elimination rather than tissue retention as such. Importantly, blood retained substantial activity (17 ± 9%ID/g) at the same time point, indicating that a notable fraction of the radiolabeled construct remained in circulation for at least up to 90 min after administration. From a translational BNCT perspective, sustained blood residence may be advantageous because the delivery of macromolecular boron carriers to solid tumors can be limited by perfusion and vascular permeability. Prolonged circulation may therefore increase the opportunity for tumor accumulation, extravasation, and retention, particularly in heterogeneous stroma-rich tumors. At the same time, a high circulating boron concentration at the time of neutron irradiation could increase the dose delivered to blood and normal tissues, thereby reducing the tumor-to-background ratio. The progressive urinary excretion observed in the present study may help decrease systemic exposure at later time points. However, the optimal administration-to-irradiation interval cannot be determined from PET data derived from healthy animals alone and will require correlation between PET imaging and direct boron quantification in blood, tumor, and relevant normal tissues.

Furthermore, uptake in major clearance and mononuclear phagocyte system (MPS) organs, including liver (4 ± 2%ID/g) and spleen (2 ± 1%ID/g), was low, with no marked accumulation in lung (4 ± 2%ID/g) or brain (0.3 ± 0.1%ID/g). Similarly, SUV_mean_ (0.42 ± 0.13) and AUC_0–90 min_ (45 SUV·min) values from liver TAC analysis (Figure 8E) also remained low over the scan duration, supporting minimal hepatobiliary clearance and limited MPS sequestration. Indeed, low hepatic and splenic retention is an encouraging feature for a macromolecular BNCT carrier, suggesting limited nonspecific trapping and potentially reducing off-target boron accumulation in dose-limiting organs. Importantly, low activity was also observed in oral mucosal tissues (2 ± 1%ID/g) and healthy tongue (1 ± 0.3%ID/g), indicating minimal nonspecific uptake of [^68^Ga]­Ga-DOTA-PBMM-*m*CB_20_ in the surrounding head-and-neck tissues. This low-background profile is practically important for BNCT applications, particularly in HNSCC, where uptake in normal mucosa and adjacent healthy tissues could complicate irradiation dose planning and thereby reduce the therapeutic efficacy.

Furthermore, previous studies have shown that nanoscale boron delivery systems, including liposomal, polymeric, and inorganic nanoparticles, can achieve favorable tumor accumulation and tumor-to-background ratios in selected tumor models.^50–52^ However, the reported outcome is highly dependent on the carrier composition and physicochemical properties, targeting strategy, tumor model used, administered dose, and biodistribution time point. These studies indeed support the potential of nanoscale boron delivery but do not yet provide a reliable basis for predicting the *in vivo* performance of the present star polymer-carborane conjugate. Therefore, its tumor boron concentration and corresponding tumor-to-normal tissue and tumor-to-blood ratios must be established experimentally in relevant tumor-bearing models.

In general, PET imaging and biodistribution data in healthy mice provide a baseline pharmacokinetic profile for the PET-trackable star polymer-carborane conjugate with three key features: (i) predominant renal elimination, (ii) a persisting blood pool activity at 90 min p.i., and (iii) minimal nonspecific accumulation in MPS organs and oral mucosa. Therefore, the presently reported modular PBMM-EIP scaffold offers clear and straightforward pathways to tune *in vivo* behavior by macromolecular engineering, for example, by adjusting monomer composition, number of arms, or changing architecture and molar mass to modulate residence time and/or introducing targeting motifs (e.g., small molecule and peptide-derived ligands) to promote tumor-selective uptake, while maintaining PET imaging capability for whole-body distribution monitoring and BNCT timing optimization between administration and neutron irradiation. However, the present *in vivo* evaluation was limited to healthy animals and a tracer-level dose selected for pharmacokinetic characterization. Consequently, these data do not establish tumor accumulation, absolute tumor boron concentration, tumor-to-background ratio, or optimal irradiation time.

## Conclusion

In this study, we developed a modular four-arm star poly­(2-oxazoline)-based platform to address key limitations of current boron delivery agents, particularly limited boron payloads and formulation constraints. A well-defined, alkyne-bearing star polymer (PBMM-EIP) was successfully synthesized by CROP with narrow dispersity and efficiently functionalized with boron-rich carborane clusters via CuAAC to generate a PBMM-*m*CB_20_-EIP conjugate with a high boron payload and excellent aqueous solubility. The achievable boron concentration in water substantially exceeded that of, for example, the clinically used BPA-fructose complex. PBMM-*m*CB_20_-EIP was better tolerated in CAL 27 and FaDu HNSCC cells while exhibiting higher cell-associated boron levels than those of BSH after 24 h of incubation. In healthy mice, PET imaging and *ex vivo* biodistribution revealed persistent blood circulation at 90 min alongside low nonspecific uptake in major organs and oral mucosa. Together, these results establish a chemically defined, water-soluble, PET-trackable star POx-carborane conjugate with a high boron payload and favorable baseline biodistribution. Although tumor-selective boron delivery and therapeutic BNCT efficacy remain to be investigated, the platform provides a promising foundation for future studies in HNSCC tumor-bearing models and for the modular optimization of circulation time and tumor selectivity.

## Supplementary Material





## Notes

Dynamic PET/CT scan (MP4)
Additional experimental details, extended methods, and full characterization data, such as ^1^H, ^1^H DOSY, ^13^C, ^19^F, ^11^B NMR spectra, SEC chromatograms, table, and supporting figures (PDF)
