# Supplementary material for: PET-Trackable Carborane-Conjugated Star-Shaped Poly(2-Oxazoline) Scaffold with High Boron Payload and Improved Aqueous Solubility for Potential Application in Boron Neutron Capture Therapy
Source: Biomacromolecules. 2026 Jul 14;27(8):5558–69. doi: 10.1021/acs.biomac.6c00871 (PMC13463551; doi:10.1021/acs.biomac.6c00871)
Supplement: Supplementary file 1 [file bm6c00871_si_002.pdf]

Supporting Information

PET-Trackable Carborane-Conjugated Star-Shaped Poly(2-Oxazoline) Scaffold with High Boron Payload and Improved Aqueous Solubility for Potential Application in Boron Neutron Capture Therapy

Yuquan Chi, Diana Barakhtii, Alessia Centanni, Vladimir Aseyev, Filip S. Ekholm, Mirkka Sarparanta, Robert Luxenhofer*, and Surachet Imlimthan*

Department of Chemistry, University of Helsinki, FI-00014 Helsinki, Finland

**Correspondences to* robert.luxenhofer@helsinki.fi and surachet.imlimthan@helsinki.fi

Table of contents

Section 1: General information (chemicals, solvents, analytical techniques and instrumentation)

Section 2: Cell culture and animal procedures

Section 3: Synthetic protocols and general procedures

Section 4: Supporting table and figures

Section 5: References

Section 1: General information

Chemicals and solvents

2-methyl-2-oxazoline (MeOx) and trifluoromethanesulfonic anhydride (Tf_2O) were obtained from abcr GmbH (Karlsruhe, Germany). 4-aminobutyl-DOTA-tris(*t*-butyl ester) was acquired from Macrocyclics (Plano, TX, USA). The sodium borocaptate (BSH) was acquired from Kat-Chem Kft. (Budapest, Hungary), while *meta*-carborane were obtained from Katchem spol. s r. o. (Prague, Czech Republic). *n*-Butyllithium (*n*-BuLi, 1.6 M in hexane) and trifluoroacetic acid were purchased from Thermo Scientific (Waltham, MA, USA). Additional reagents, including methanol (MeOH), diethyl ether (Et_2O), acetonitrile (ACN), dichloromethane (DCM), pyridine, phosphorus pentoxide (P_2O_5), propargyl bromide, copper bromide (CuBr), pentamethyldiethylenetriamine (PMDETA), tetramethylethylenediamine (TMEDA), N,N-diisopropylethylamine (DIPEA), hexafluorophosphate azabenzotriazole tetramethyl uronium (HATU), ethyl isonipecotate, calcium hydride (CaH_2), N-iodosuccinimide (NIS), triphenylphosphine (PPh_3), oxetane, sodium azide, and pentaerythritol were purchased from Sigma-Aldrich (St. Louis, MO, USA). Sephadex LH-20 gel for column purification was purchased from Sigma-Aldrich. Silica gel 40 for column purification was purchased from VWR International (Radnor, PA, USA). Aluminum sheets precoated with silica gel 60 F_{254} for TLC were purchased from Merck KGaA (Darmstadt, Germany), and separations were visualized by staining with 2 wt.% $PdCl_2$ (A2B Chem, San Diego, CA, USA) solution in MeOH and heating. Deuterated solvents for NMR spectroscopy were purchased from Eurisotop (Paris, France).

MeOx was distilled over CaH_2 into a Schlenk flask and stored under a nitrogen atmosphere. ACN was distilled over P_2O_5 into a Schlenk flask and stored under nitrogen. Pyridine and THF were distilled over CaH_2 into Schlenk flasks containing activated 4 Å molecular sieves and

stored under nitrogen atmosphere. All solvents used for polymerizations had a water content below 30 ppm, as determined by Karl-Fischer titration (Mettler Toledo Titrator Compact C20SX). All reagents and glassware were dried prior to polymerization. Polymerizations were carried out in a flame-dried Schlenk flask under a nitrogen atmosphere.

Size exclusion chromatography (SEC)

SEC measurements were carried out on an Agilent 1260 Infinity II GPC-SEC-MDS system equipped with a 1260 Infinity II isocratic pump, degasser, vial sampler, multicolumn thermostat (MCT), variable wavelength detector (standard flow cell), fluorescence detector, and an Agilent triple-detection suite comprising a 1260 GPC/SEC MDS refractive index detector, MDS viscometer detector, and MDS dual-angle light scattering detector. Separation was achieved using Waters Styragel columns (HT5, HR4, and HR2; 300 mm) at 40 °C column oven. The mobile phase was HPLC-grade DMF (Sigma-Aldrich, Steinheim, Germany) containing analytical grade LiBr (1 g/L, Merck, Darmstadt, Germany) at a flow rate of 0.8 mL/min. The calibration curve was prepared using poly(methyl methacrylate) (PMMA) standards (Polymer Standards Service, Mainz, Germany). Samples were dissolved in the mobile phase and filtered through a 0.22-µm PTFE syringe filter prior to injection.

Nuclear magnetic resonance (NMR)

^{1}H NMR spectra were obtained using a Bruker Avance III spectrometer (500.13 MHz) or a Bruker Avance Neo spectrometer (400.14 MHz). Measurements were carried out at 25 °C without sample spinning, using a 30° flip angle and a recycle delay of 1.5 sec. ^{13}C NMR spectra was recorded on a Bruker Avance Neo spectrometer (125.76 MHz) at 25 °C. ^{11}B NMR spectra were recorded on a Bruker Avance Neo spectrometer (160.46 MHz) at 25 °C using power-gated decoupling. ^{19}F NMR spectra were obtained using a Bruker Avance Neo spectrometer (376.47 MHz). For the diffusion-ordered NMR spectroscopy (DOSY), the spectra were recorded on a

Bruker Avance III spectrometer (500.13 MHz) at 25 °C (without temperature calibration) using the ledbpgp2s pulse sequence without sample rotation. All measurements were conducted in deuterated methanol. The diffusion gradient was linearly incremented in 6 steps from 2% to 98%. The diffusion time (d20) was set to 0.05 sec, and the gradient pulse length (p30) was 3000 μs. Data were acquired with 8 scans and a recycle delay of 5 sec.

Mass spectrometry (MS)

Mass spectra were recorded on a Bruker microTOF spectrometer equipped with an atmospheric pressure chemical ionization (APCI) source operating in positive-ion mode. Samples were introduced using a mixture of dichloromethane (DCM), isopropanol (IPA), and in some cases formic acid (FA). The instrument parameters were optimized as follows: the capillary exit and skimmer 1 were maintained at 90 V and 35 V, respectively; the hexapole RF was set to 90 Vpp. The transfer time was established at 25 μs with a pre-pulse storage time of 7 μs. For ionization, the heater temperature was set to 250 °C and the vaporizer temperature was maintained at 450 °C.

Dynamic light scattering (DLS)

DLS measurements were conducted using a Brookhaven Instruments Co. (BIC) BI-200SM goniometer equipped with a BIC-TurboCorr digital pseudo-cross-correlator and a BI-CrossCorr detector (two BIC-DS1 detectors). A Sapphire 488-100 CDRH laser (Coherent GmbH, 488 nm, 10–50 mW) was used as the light source. Measurements were collected at a scattering angle of 90° and 25 °C using a 0.4 mm detector pinhole. Samples were equilibrated for 30 min before measurement. For sample preparation, unmodified star polymer and star polymer-carborane conjugate were dissolved in dH_2O at concentrations of 10 g/L and 1 g/L, respectively, and filtered through 0.45-μm PTFE membranes directly to dust-free cuvettes.

Fourier transform infrared (FTIR) spectroscopy

IR-spectra were recorded on a Shimadzu IR Tracer-100 Fourier transform infrared spectrometer over 4000–650 cm^{-1} at ambient temperature. Each spectrum was acquired using 64 scans.

Section 2: Cell culture and animal procedures

Cell culture materials

Cell culturing flasks and multi-well plates were purchased from VWR International (Radnor, PA, USA). Dulbecco's Modified Eagle's Medium (DMEM; for CAL 27), Eagle's Minimum Essential Medium (EMEM; for FaDu), GlutaMAX (100×), sodium pyruvate (100 mM), fetal bovine serum (FBS), Penicillin-Streptomycin (P/S, 10,000 U/mL), TrypLE™ Express, Dulbecco's phosphate-buffered saline (1× DPBS, pH 7.4), and sterile bottles equipped with 0.22-μm PES filter membranes were obtained from Gibco (Life Technologies, Carlsbad, CA, USA). In general, complete culture media were supplemented to the final concentrations of 1× GlutaMax, 1 mM sodium pyruvate, 10% (v/v) FBS, and 100 U/mL P/S and aseptically filtered before use. The CellTiter-Glo® luminescent cell viability assay was purchased from Promega Corporation (Madison, WI, USA). Cell counting was done using an EVE™ automated cell counter and counting kit (NanoEnTek, Guro-gu, Seoul, Korea). Cells were maintained at 37 °C in a humidified cell incubator (NuAire, Plymouth, MN, USA) with 5% CO_2 atmosphere.

Cell-associated boron quantification

For boron quantification, cells were pelleted again, and the supernatant was removed. The pellet was digested in 0.5 mL of 50% (v/v) nitric acid (HNO_3) at 80 °C and 300 rpm for 3 h. Digests were cooled to RT and diluted with 3.5 mL of dH_2O. Samples were filtered through 0.22-μm membrane, after which 50 μL of cesium ionization buffer (Agilent Technologies, Santa Clara, CA, USA) and 25 μL of 100 ppm scandium internal standard (ROMIL, Cambridge, UK) in 5% HNO_3 were added. Boron content was measured using microwave plasma atomic emission spectroscopy (MP-AES 4200, Agilent Technologies, Santa Clara, CA, USA). The detection wavelengths for boron and scandium are 249.772 and 361.383 nm, respectively.

Quantification was conducted using external calibration standards prepared to match the boron concentrations used during cell incubation. Cell-associated boron was expressed as absolute boron content (ng), normalized to 1×10^6 cells.

Animal experimentation

Animal studies were conducted in healthy female SWISS Rj:SWISS mice (weighed 22–24 g, Janiver Laboratories, Le Genest-Saint-Isle, France). Animals were housed in conventional polysulfone cages with aspen bedding (Tapvei®, Harjumaa, Estonia) and environmental enrichments (e.g., nesting material and polysulfone tunnels). Standard chow (Teklad 2019C diet pellets, Envigo, Huntingdon, UK) and tap water were provided *ad libitum*. The animal housing unit was maintained on a 12 h light/12 h dark cycle at 21 ± 1 °C and 55 ± 15% relative humidity.

Section 3: Synthetic protocols and general procedures

Synthesis of four-arm initiator pentaerythritol tetrakistriflate (PE(OTf)$_4$)

Synthesis of PE(OTf)$_4$ was carried out based on a previously published protocol with minor modifications.[1] Pentaerythritol (504 mg, 3.7 mmol, 1 eq.) was dissolved in a mixture of dry pyridine (2.5 mL) and dry ACN (4 mL) in a 100 mL three-necked round-bottomed flask and cooled to 0 °C in an ice bath. Triflic anhydride (5 g, 17.76 mmol, 4.8 eq.) was added dropwise over about 15 min. The reaction mixture was allowed to warm to room temperature and stir for 3 h, after which 1 M HCl (20 mL) was added. The reaction flask was further cooled in an ice bath to allow product precipitation (orange color). The precipitate was filtered by Büchner funnel and washed with cold water. Then, recrystallization from hot ACN/H_2O (1:1 v/v) mixture yielded white crystals (1.75 g, 71% yield).

Synthesis of 2-butyne-2-oxazoline monomer (BynOx)

The compound was synthesized according to a previously published procedure with slight modifications.[2, 3] Tetraacetylethylenediamine (TMEDA, 3.68 g, 31.7 mmol, 1 eq.) was dissolved in dry THF (150 mL) in a dried, nitrogen-flushed Schlenk flask and cooled to –78 °C. After 30 min cooling, *n*-BuLi (20 mL, 1.6 M in hexanes, 1 eq.) was added, and the mixture was stirred for an additional 60 min. Subsequently, 2-methyl-2-oxazoline (MeOx, 2.7 g, 31.7 mmol, 1 eq.) was then added dropwise, and the reaction was stirred for 2 h at −78 °C. Propargyl bromide (3.77 g, 31.7 mmol, 1 eq.) was added, after which the reaction mixture was allowed to warm to room temperature and stirred overnight. After 24 h, the reaction was quenched with MeOH (75 mL, 1.85 mol, 58 eq.), and the solvents were removed under reduced pressure. The residue was dissolved in DCM (60 mL) and washed with saturated aqueous $NaHCO_3$ solution (3 × 50 mL). The aqueous phase was extracted with DCM (2 × 70 mL), and the combined organic phases washed with distilled water (3 × 120 mL), dried over anhydrous Na_2SO_4, and

concentrated under reduced pressure. The crude product was purified by distillation under reduced pressure (10 mbar, oil bath 110–120 °C, actual temperature 64 °C) to obtain the product as white, needle-like crystals (0.9 g, 23% yield).

Deprotection of star p(BynOx$_5$-*ran*-MeOx$_5$)-*b*-pMeOx$_{60}$-isonipecotate (PBMM-COOH)

Aqueous NaOH (1 M, 1 mL) was added to PBMM-EIP (8 µmol, 1 eq.) in MeOH (1 mL) while stirring. The reaction mixture was heated to 50 °C and stirred for 2 h. After completion, the solvents were removed under reduced pressure. The residue was dissolved in dH_2O and carefully neutralized to pH 6–7 with 1 M HCl. The polymer solution was purified by dialysis (MWCO 1 kDa) against dH_2O for 24 h and then lyophilized to obtain a white powder product.

Synthesis of boron cluster conjugation block: 1-(3-azopropyl)-1,7-dicarba-*closo*-dodecaborane (*m*CB-Pr-N_3) *via* a three-step reaction

Step 1: 1-(3-hydroxypropyl)-1,7-dicarba-*closo*-dodecaborane

The protocol reported by Agarwal *et al.*[4] was followed. In brief, *meta*-carborane (2 g, 13.86 mmol, 1 eq.) was dissolved in 20 mL of anhydrous THF and deprotonated through the dropwise addition of *n*-BuLi (2.5 M in hexane, 6.1 mL, 15.25 mmol, 1.1 eq.) at −78 °C. The reaction mixture was warmed to 0 °C, and a solution of oxetane (904 µL, 13.86 mmol, 1 eq.) in anhydrous THF (5 mL) was added. The reaction mixture was allowed to stir overnight. The reaction was quenched by the addition of saturated aqueous NH_4Cl solution (30 mL), and the mixture was extracted with EtOAc (3 × 40 mL). The combined organic layers were dried with anhydrous Na_2SO_4 and concentrated under reduced pressure. The crude product was purified by column chromatography using a mixture of EtOAc:Hex (3:7) as the eluent and dried under vacuum to yield a white solid (1.1 g, 38% yield). Thin-layer chromatography (TLC) analysis provided an R_f value of 0.59 using EtOAc:Hex (3:7) as the eluent. The analytical data was in agreement with the previous report.[4]

Step 2: 1-(3-iodopropyl)-1,7-dicarba-*closo*-dodecaborane

1-(3-hydroxypropyl)-1,7-dicarba-*closo*-dodecaborane (1.07 g, 5.24 mmol, 1 eq.) from step 1 was dissolved in dry DCM (15 mL), and the solution was cooled to 0 °C. Subsequently, triphenylphosphine (PPh_3, 1.65 g, 6.29 mmol, 1.2 eq.) and *N*-iodosuccinimide (NIS, 1.47 g, 6.55 mmol, 1.25 eq.) were slowly added with stirring. The ice bath was then removed, and the reaction mixture was stirred overnight at RT. The reaction progress was monitored using TLC method described earlier. After sufficient conversion, hexane (30 mL) was added to precipitate the formed triphenylphosphine oxide. The resulting yellow mixture was filtered through a silica pad, rinsed with hexane (3 × 15 mL), and the filtrate was concentrated under reduced pressure at 40 °C. The crude product was purified by column chromatography using EtOAc:Hex (3:7) as the eluent and dried under vacuum to yield a viscous, opaque liquid (1.47 g, 90% yield). TLC analysis provided an R_f value of 0.59 using EtOAc:Hex (3:7) as the eluent. The analytical data was in agreement with the previous report.[4]

Step 3: 1-(3-azopropyl)-1,7-dicarba-*closo*-dodecaborane (*m*CB-Pr-N_3)

Protocols for the synthesis of azidopropyl *meta*-carborane have been published previously.[4] Briefly, 1-(3-iodopropyl)-1,7-dicarba-*closo*-dodecaborane (1.47 g, 4.7 mmol, 1 eq.) from step 2 was dissolved in anhydrous DMF (20 mL), and NaN_3 (0.92 g, 14.1 mmol, 3 eq.) was then added. The reaction mixture was stirred overnight at RT. The crude product was purified by silica gel column chromatography using EtOAc:Hex (3:7) as the eluent and dried under vacuum to obtain a colorless liquid (1 g, 94% yield). TLC analysis of the product provided an R_f value of 0.87 using EtOAc:Hex (3:7) as the eluent.

Synthesis of deprotected star polymer-carborane conjugate (PBMM-*m*CB$_{20}$-COOH) *via* CuAAC

PBMM-COOH (37 mg, 2.8 μmol, 1 eq.), *m*CB-Pr-N_3 (15.3 mg, 67.2 μmol, 24 eq.), PMDETA (23.3 mg, 134.3 μmol, 48 eq.), and DMF (2 mL) were added to a 5 mL flame-dried Schlenk flask. The reaction mixture was degassed by bubbling N_2 for 20 min. The click reaction was initiated by addition of CuBr (19.3 mg, 134.3 μmol, 2 eq. relative to azide), and the reaction solution was stirred for 24 h at room temperature. Then, the mixture was concentrated under vacuum and purified by SEC on Sephadex LH-20 column in MeOH. The solvent was removed under a stream of air, and the product was lyophilized to obtain a white powder (30 mg, 60% yield).

Synthesis of star polymer-carborane conjugate (PBMM-*m*CB$_{10}$-EIP) *via* CuAAC

PBMM-EIP (19.6 mg: SEC-based mass, 1.3 μmol, 1 eq.), *m*CB-Pr-N_3 (3.5 mg, 15.6 μmol, 12 eq.), PMDETA (5.4 mg, 31.2 μmol, 24 eq.), and DMF (1 mL) were added to a 5 mL flame-dried Schlenk flask. The reaction mixture was degassed by bubbling N_2 for 20 min. The click reaction was initiated by addition of CuBr (4.5 mg, 31.2 μmol, 2 eq. relative to azide), and the reaction was stirred for 24 h at room temperature. Then, the mixture was concentrated under vacuum and purified by SEC on Sephadex LH-20 column in MeOH. The solvent was removed under a stream of air, and the product lyophilized to obtain a white powder (19.3 mg, 92% yield).

Quality control of gallium-68-labeled DOTA-PBMM-*m*CB$_{20}$

For quality control, an aliquot (2 μL) of crude and purified product was spotted onto instant thin-layer chromatography impregnated with silica gel strips (iTLC-SG, Agilent Technologies, Santa Clara, CA, USA). Chromatography was developed using 20 mM citric acid (pH 5) as the mobile phase, in which free gallium-68 migrated with the solvent front ($R_f \approx 1$) while [^{68}Ga]Ga-DOTA-PBMM-*m*CB$_{20}$ remained at the origin ($R_f \approx 0$). The iTLC-SG strips were imaged by autoradiography (Fujifilm Fluorescent Image Analyzer FLA-5100, Fuji Film Photo, Tokyo,

Japan), and radiochemical purity (RCP) of the product was quantified by Aida software (v5.0 SP3, Elysia-Raytest GmbH, Straubenhardt, Germany). The purified [^{68}Ga]Ga-DOTA-PBMM-*m*CB_{20} was formulated in sterile 0.9% isotonic saline for *in vivo* studies.

Section 4: Supporting table and figures

Table S1. Characterization of both before and after CuAAC of *m*CB-Pr-N_3 to PBMM-EIP

Sample	$M_{n, theo}$ a) (kg mol^{-1})	$M_{n, NMR}$ b) (kg mol^{-1})	$M_{n, SEC}$ c) (kg mol^{-1})	Đ d)	Yield (%)
PBMM-EIP	25	N/A	15	1.15	87%
PBMM-*m*CB_{20}-EIP	30	N/A	13	1.23	70%

a) Calculated from $[M]_0/[I]_0$ and for 100% conversion for polymers; b) N/A: M_n could not be reliably determined by ^{1}H NMR due to overlapping signals and incomplete termination efficiency; c) Determined by SEC (DMF + LiBr, PMMA standard); d) Đ = M_w/M_n calculated from SEC analysis.

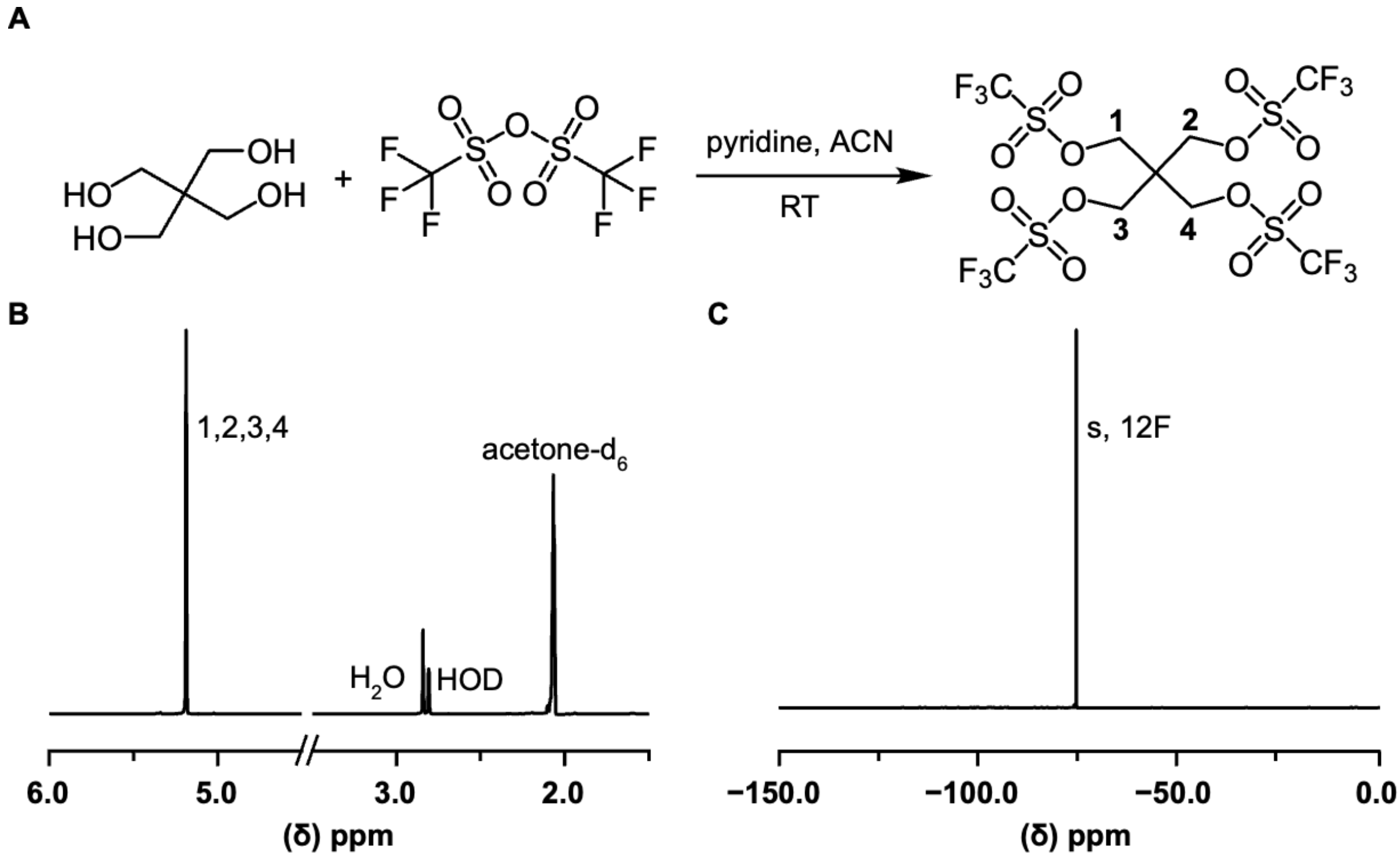

Figure S1. Reaction scheme and characterization of pentaerythritol tetrakistriflate. (**A**) Synthetic route of pentaerythritol tetrakistriflate. (**B**) ^{1}H NMR spectrum in acetone-d_6. (**C**) ^{19}F NMR spectrum in acetone-d_6.

^{1}H NMR (acetone-d$_6$, 500.13 MHz): δ [ppm]: 5.17 (s, 8H, H^1).

^{19}F NMR (acetone-d$_6$, 376.47 MHz): δ [ppm]: −75.1 (s, 12F).

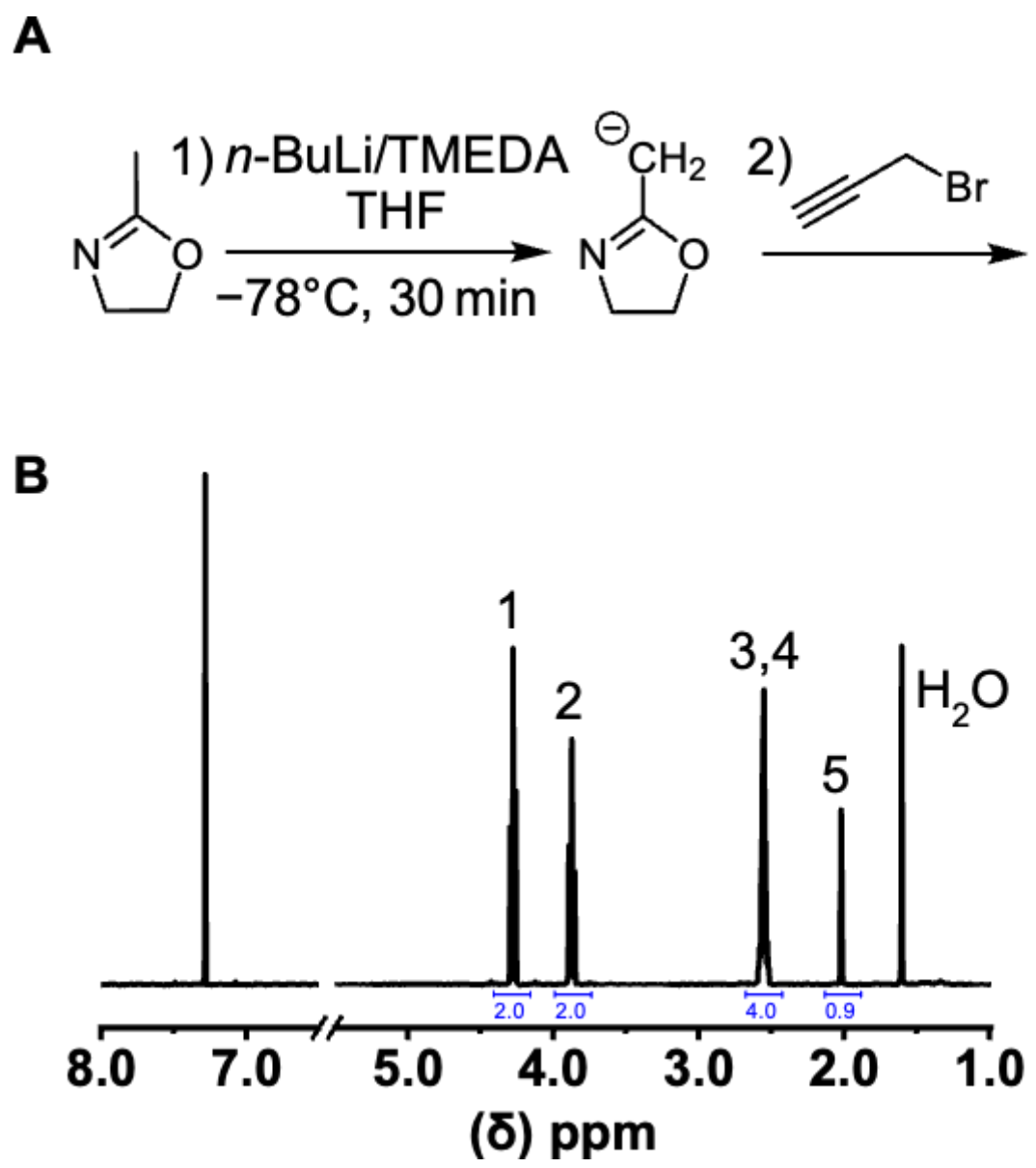

Figure S2. (**A**) Synthetic route of BynOx and (**B**) ^{1}H NMR spectrum in $CDCl_3$ of 2-butyne-2-oxazoline monomer.

^{1}H NMR ($CDCl_3$, 500.13 MHz): δ [ppm]: 4.25 (t, 2H, H^1), 3.85 (t, 2H, H^2), 2.52 (m, 4H, H^{3,4}), 1.99 (s, 1H, H^5). All ^{1}H NMR integrals reported hereafter are normalized to a single arm of the star polymer.

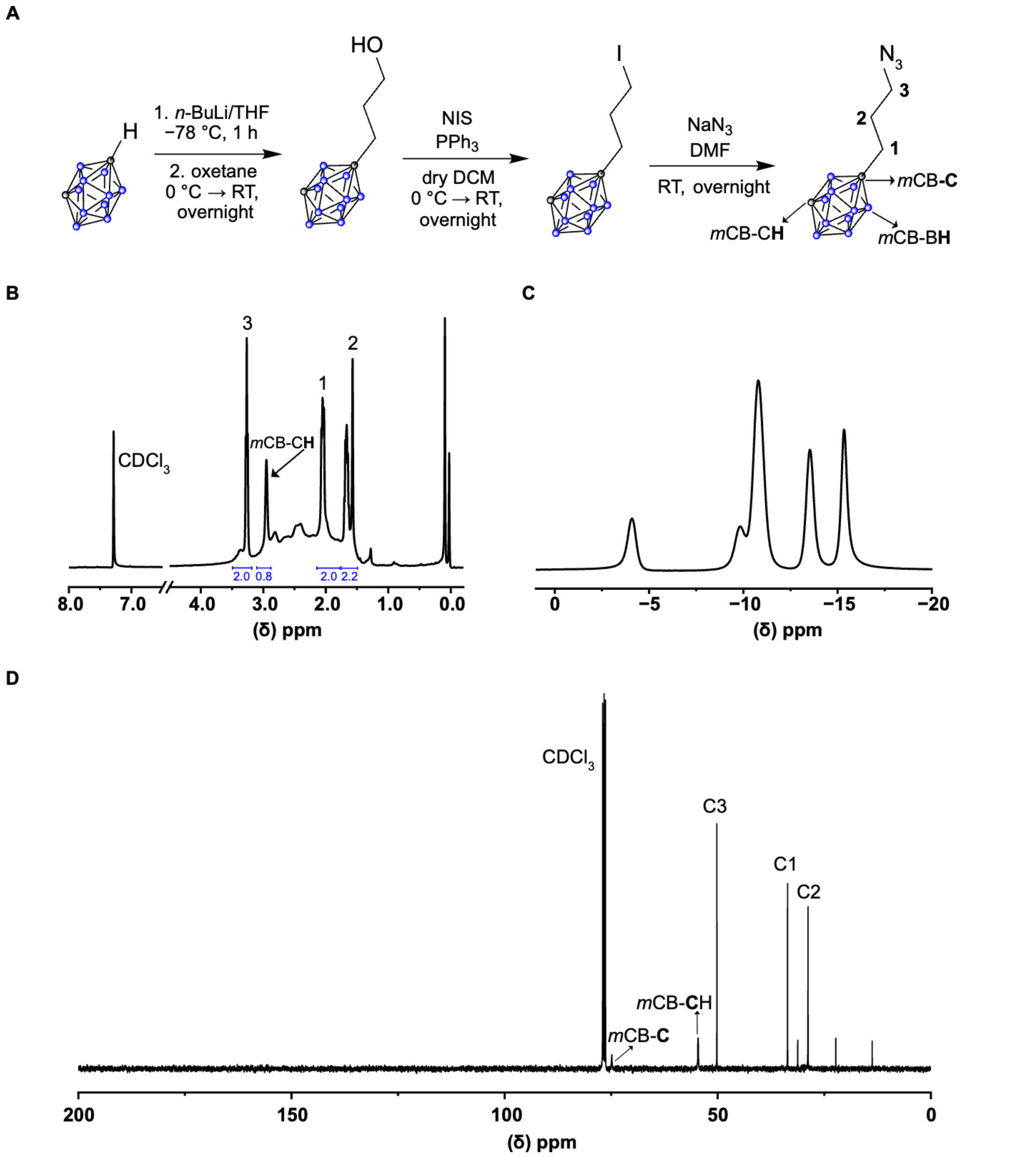

Figure S3. Synthetic routes and characterizaitons of 1-(3-azopropyl)-1,7-dicarba-*closo*-dodecaborane (*m*CB-Pr-N_3). (**A**) Synthetic routes of *m*CB-Pr-N_3. (**B**) ^{1}H NMR spectrum in $CDCl_3$. (**C**) ^{11}B NMR spectrum in $CDCl_3$. (**D**) ^{13}C NMR spectrum in $CDCl_3$.

^{1}H NMR ($CDCl_3$, 500.13 MHz): δ [ppm]: 3.26 (t, 2H, H^3), 3.11−1.50 (br m, 10H, *m*CB-B**H**), 2.90 (br s, 1H, *m*CB-C**H**), 2.02 (m, 2H, H^1), 1.64 (m, 2H, H^2).

$^{13}C\{^{1}H\}$ NMR (125.76 MHz, $CDCl_3$): δ [ppm]: 75.4 (*m*CB-**C**), 55.1 (*m*CB-**C**H), 50.8 (C^3), 34.2 (C^1), 29.4 (C^2).

$^{11}B\{^{1}H\}$ NMR (160.46 MHz, $CDCl_3$): δ [ppm]: −4.0, −9.8, −10.7, −13.5, −15.3.

HRMS: *m/z* found 202.2373 for $C_5H_{18}B_{10}N_1$ $[M – N_2 + H]^+$ (*m/z* calc. 202.2370)

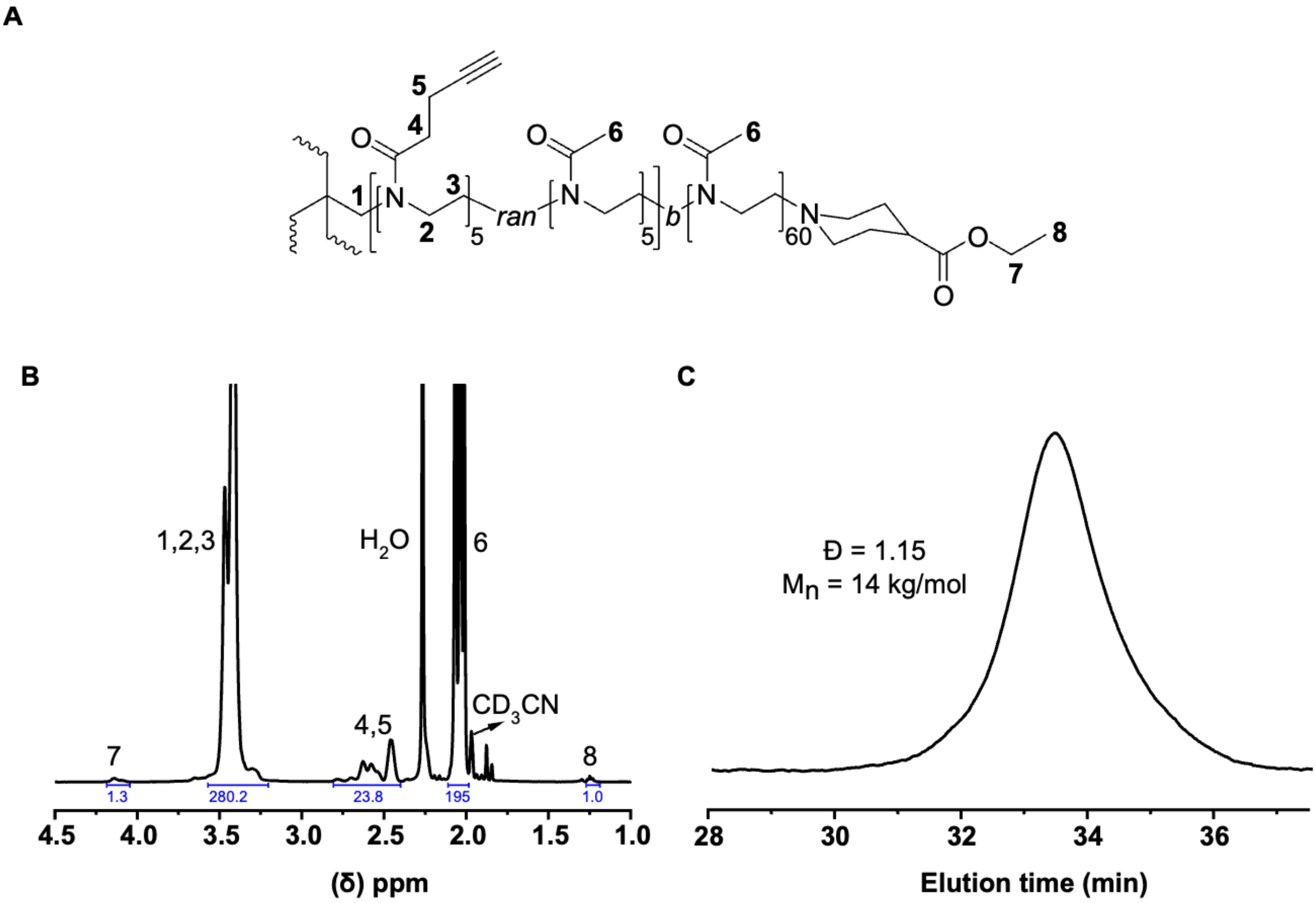

Figure S4. PBMM-EIP characterization. (**A**) Chemical structure of PBMM-EIP. (**B**) ^{1}H NMR spectrum in CD_3CN. (**C**) SEC chromatogram (RI signal, eluent: DMF containing 1g/L LiBr).

^{1}H NMR (CD_3CN, 500.13 MHz): δ [ppm]: 4.12 (q, 1H, H^7), 3.66-3.10 (br, 280H, $H^{1,2,3}$), 2.67–2.39 (br, 24H, $H^{4,5}$), 2.07–1.95 (br, 195H, H^6), 1.24 (t, 1H, H^8).

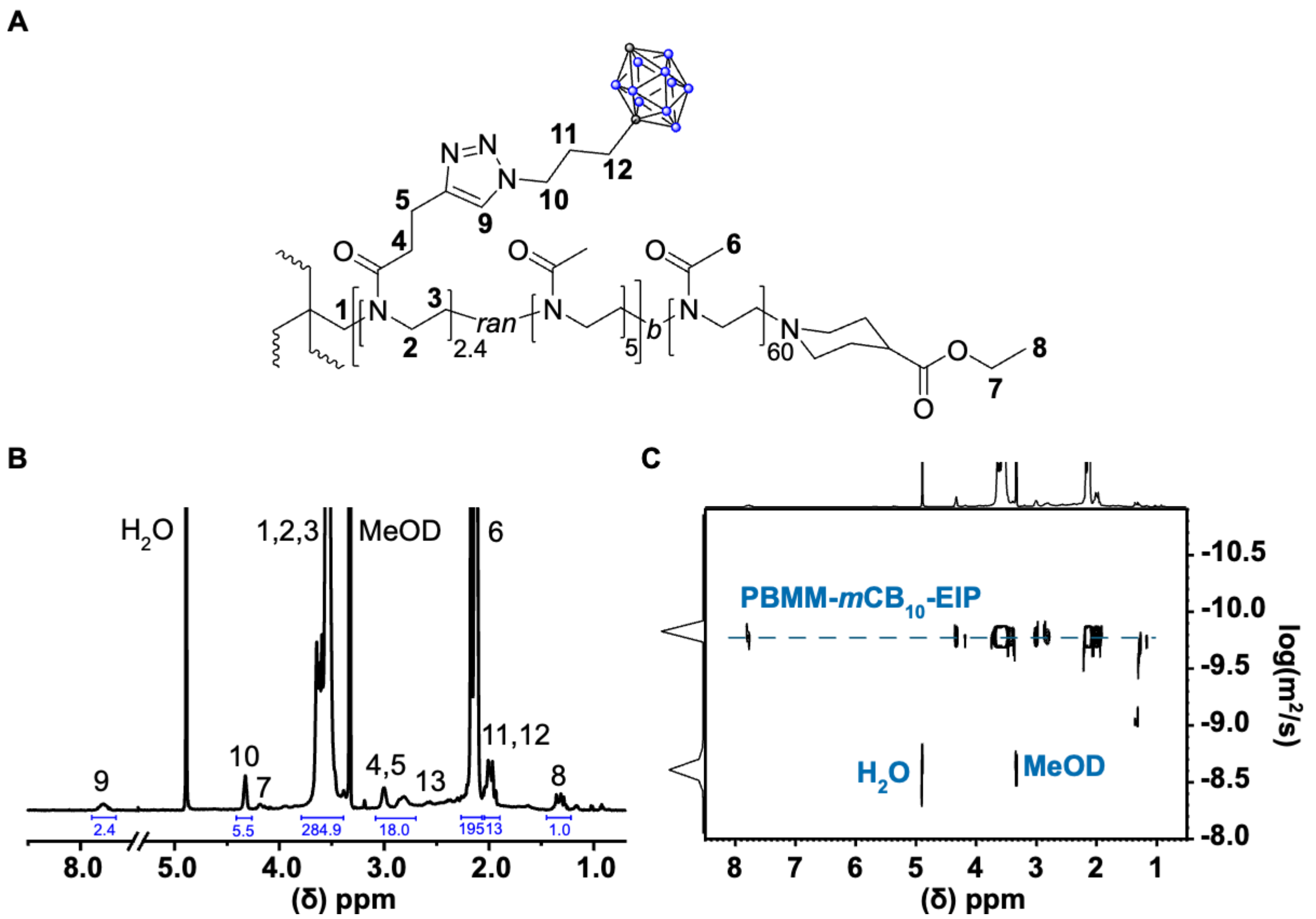

Figure S5. PBMM-mCB$_{10}$-EIP characterization. (**A**) Chemical structure of PBMM-mCB$_{10}$-EIP. (**B**) ^{1}H NMR spectrum in MeOD. (**C**) ^{1}H DOSY NMR in MeOD.

^{1}H NMR (MeOD, 500.13 MHz): δ [ppm]: 7.77 (s, 2H, H^9), 4.31 (br, 6H, H^{10}), 4.12 (q, 1H, H^7), 3.81−3.40 (br, 285H, $H^{1,2,3}$), 3.10−2.60 (br, 18H, $H^{4,5}$), 2.23−2.05 (br, 195H, H^6), 2.04−1.86 (br, 13H, $H^{11,12}$), 1.5−2.7 (br, B–H of mCB cluster), 1.29−1.26 (br, 1H, H^8).

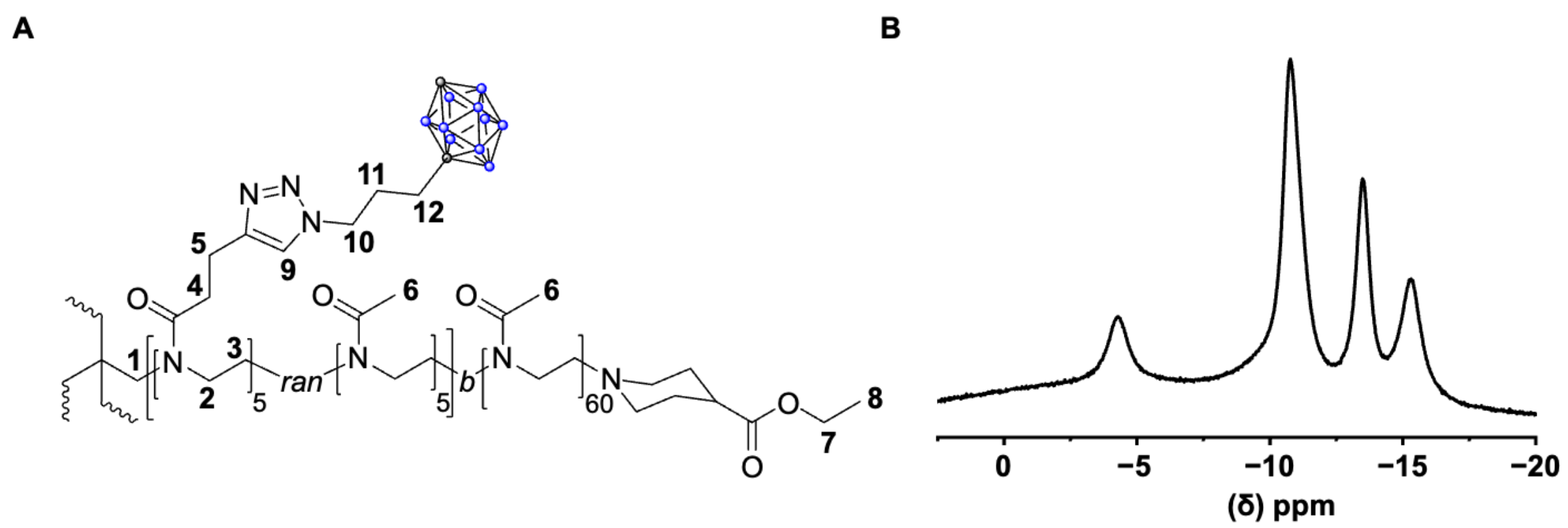

Figure S6. PBMM-*m*CB_{20}-EIP characterization. (**A**) Chemical structure of PBMM-*m*CB_{20}-EIP. (**B**) Proton-decoupled ^{11}B NMR spectrum of PBMM-*m*CB_{20}-EIP.

^{1}H NMR (MeOD, 500.13 MHz): δ [ppm]: 7.77 (s, 5H, H^{9}), 4.31 (br, 10H, H^{10}), 4.12 (q, 1H, H^{7}), 3.81−3.40 (br, 278H, $H^{1,2,3}$), 3.10−2.60 (br, 18H, $H^{4,5}$), 2.23−2.05 (br, 195H, H^{6}), 2.04−1.86 (br, 20H, $H^{11,12}$), 1.5−2.7 (br, B–H of *m*CB cluster), 1.26−1.1 (br, 1H, H^{8}).

$^{11}B\{^{1}H\}$ NMR (160.46 MHz, $CDCl_3$): δ [ppm]: −15.5, −13.6, −11.0, −4.5.

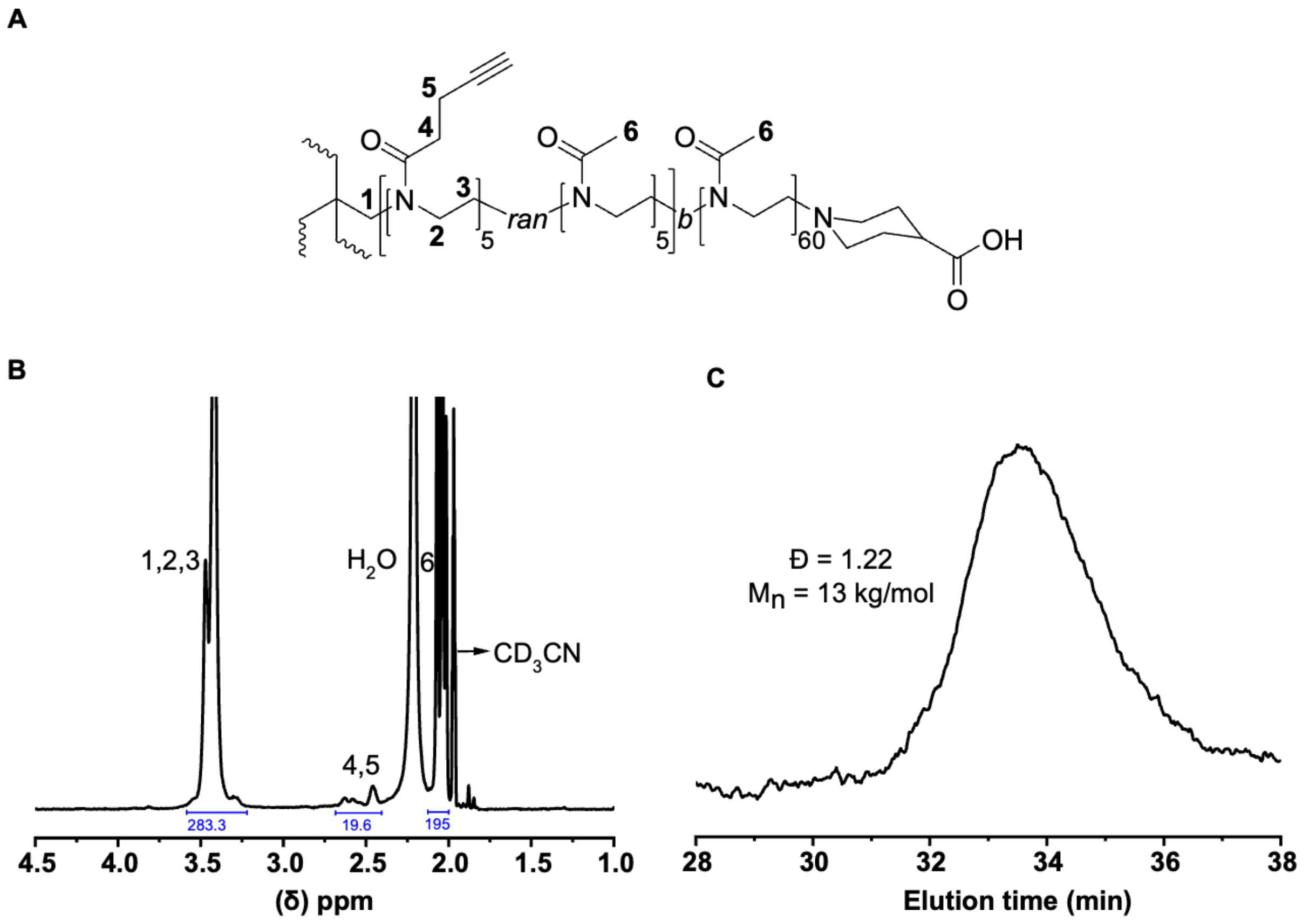

Figure S7. PBMM-COOH characterization. (**A**) Chemical structure of PBMM-COOH. (**B**) ^{1}H NMR spectrum in CD_3CN. (**C**) SEC chromatogram (RI signal, eluent: DMF containing 1 g/L LiBr).

^{1}H NMR (CD_3CN, 500.13 MHz): δ [ppm]: 3.66–3.10 (br, 283H, $H^{1,2,3}$), 2.67–2.39 (br, 20H, $H^{4,5}$), 2.07–1.95 (br, 195H, H^{6}).

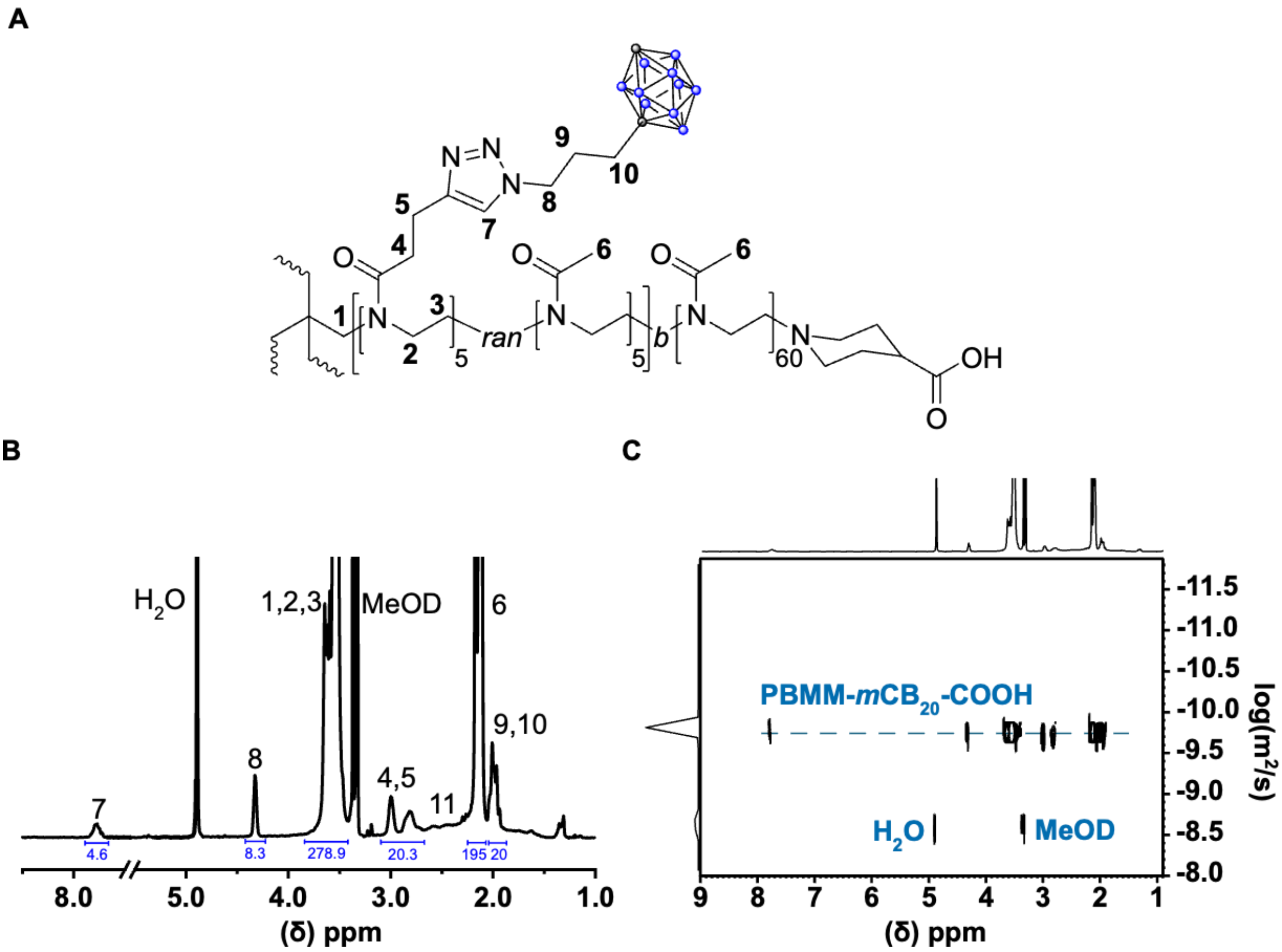

Figure S8. PBMM-*m*CB$_{20}$-COOH characterization. (**A**) Chemical structure of PBMM-*m*CB$_{20}$-COOH. (**B**) ^{1}H NMR spectrum in MeOD. (**C**) ^{1}H DOSY NMR spectrum in MeOD.

^{1}**H NMR** (MeOD, 500.13 MHz): δ [ppm]: 7.77 (s, 5H, H^{7}), 4.31 (br, 8H, H^{8}), 3.81−3.40 (br, 279H, $H^{1,2,3}$), 3.10−2.60 (br, 20H, $H^{4,5}$), 2.23−2.05 (br, 195H, H^{6}), 2.04−1.86 (br, 20H, $H^{9,10}$), 1.5−2.7 (br, B–H of *m*CB cluster).

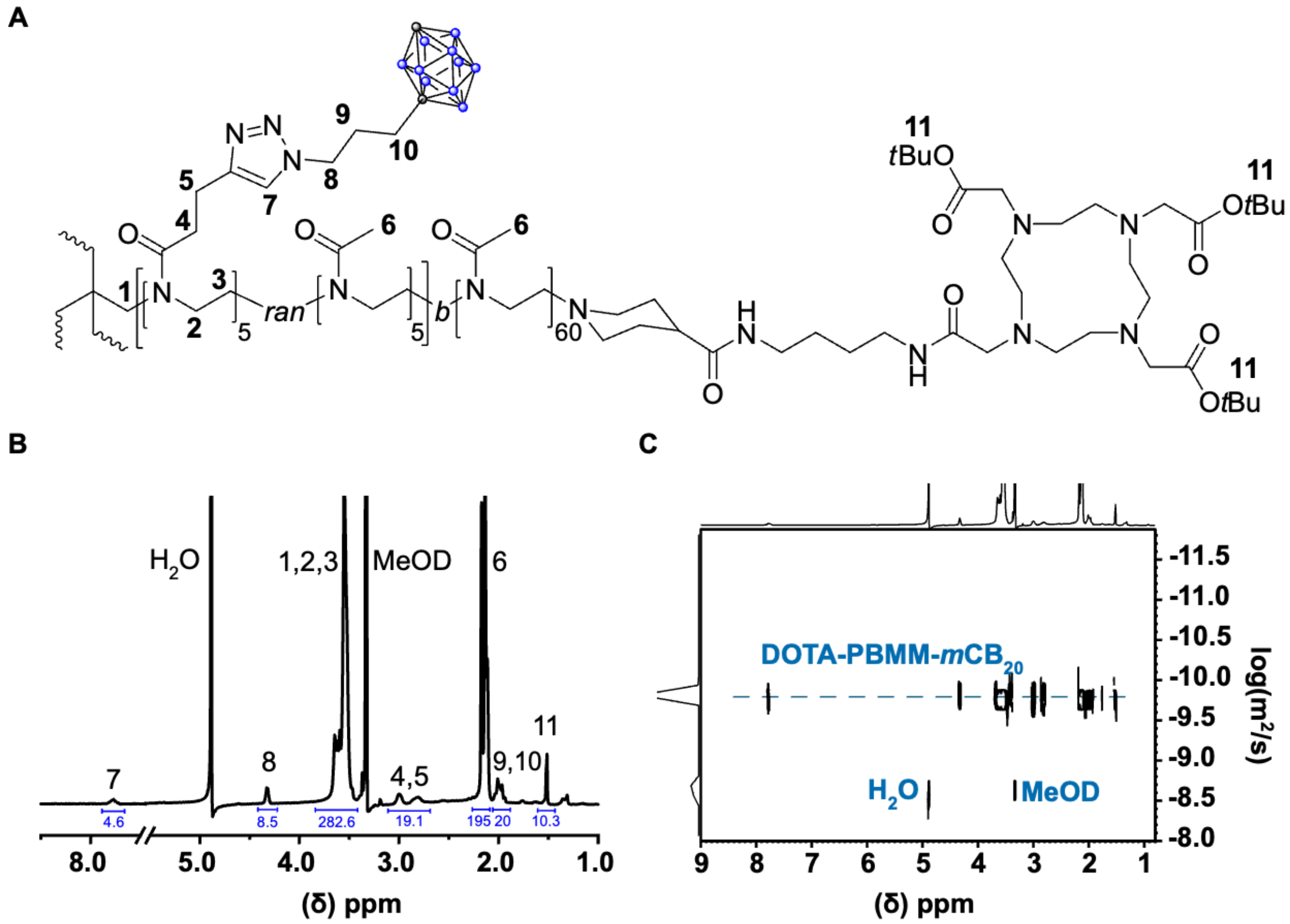

Figure S9. DOTA-PBMM-*m*CB_{20} characterization. (**A**) Chemical structure of DOTA-PBMM-*m*CB_{20}. (**B**) ^{1}H NMR spectrum in MeOD. (**C**) ^{1}H DOSY NMR spectrum in MeOD.

^{1}H NMR (MeOD, 500.13 MHz): δ [ppm]: 7.77 (s, 5H, H^7), 4.31 (br, 9H, H^8), 3.81−3.40 (br, 283H, $H^{1,2,3}$), 3.10−2.60 (br, 19H, $H^{4,5}$), 2.23−2.05 (br, 195H, H^6), 2.04−1.86 (br, 20H, $H^{9,10}$), 1.5−2.7 (br, B–H of *m*CB cluster), 1.54−1.50 (s, 10H, H^{11}).

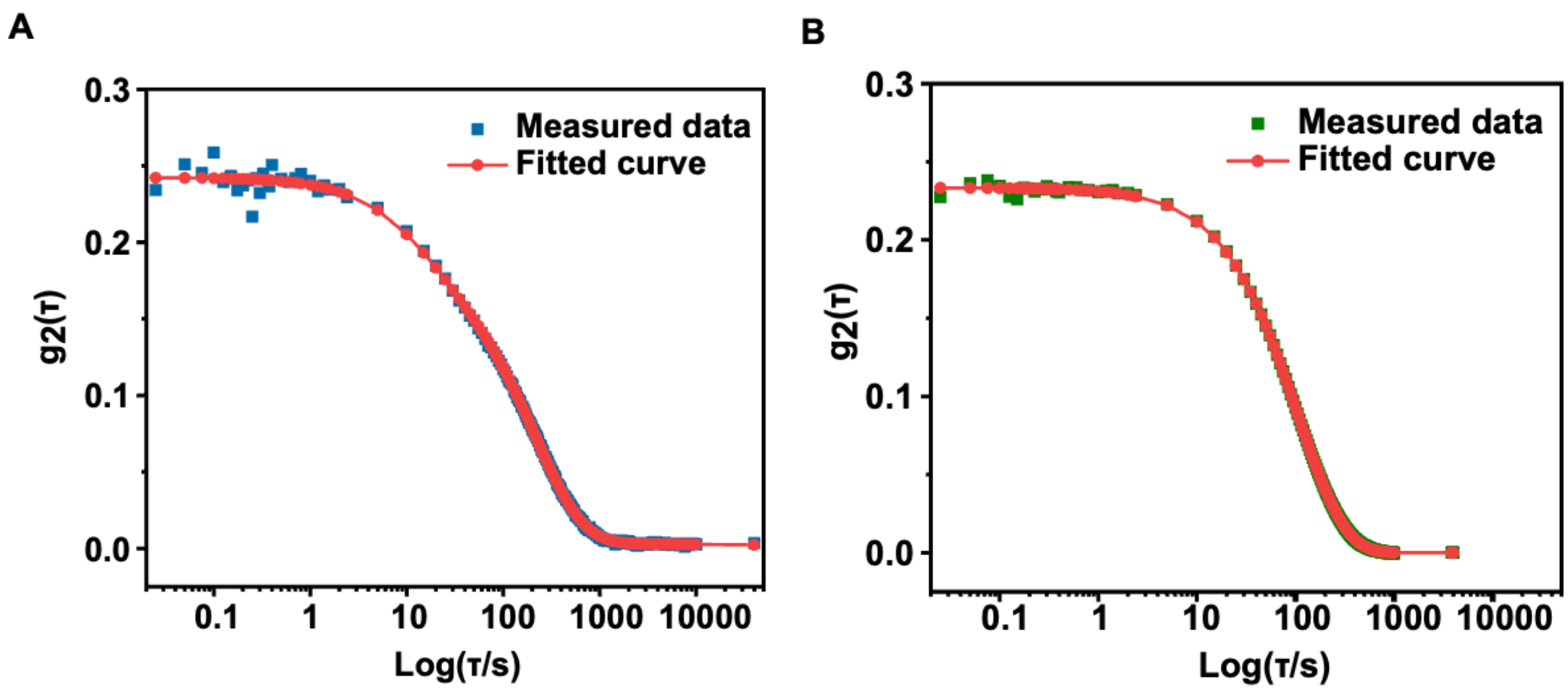

Figure S10. Pseudo-cross correlation function of the scattered light intensity of (**A**) PBMM-EIP (10 g/L) and (**B**) PBMM-*m*CB$_{20}$-EIP (1 g/L). Measurements were conducted with 0.45-µm filtered samples at a scattering angle of 90°. The fit was carried out using an inverse Laplace transform program CONTIN.

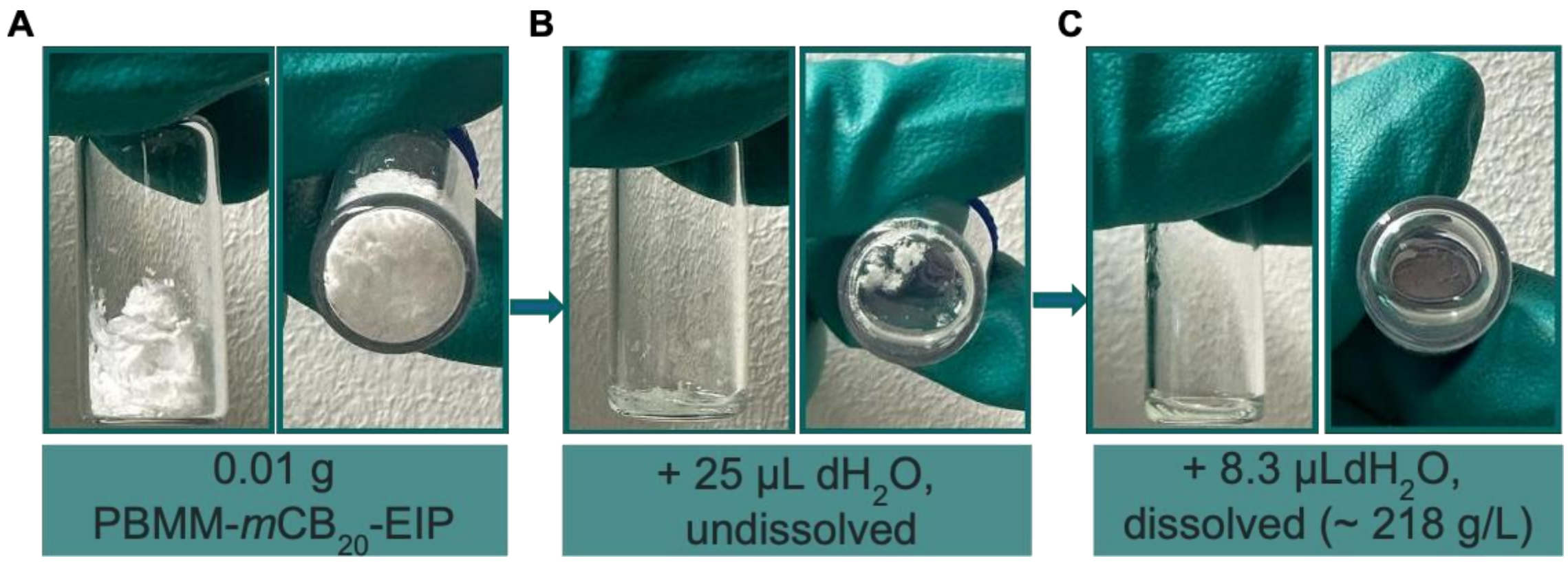

Figure S11. Visual observation of aqueous solubility of PBMM-*m*CB$_{20}$-EIP. (**A**) PBMM-*m*CB$_{20}$-EIP as a solid powder. (**B**) PBMM-*m*CB$_{20}$-EIP after addition of 25 µL of dH_2O, showing incomplete dissolution. (**C**) PBMM-*m*CB$_{20}$-EIP after addition of 8.3 µL of dH_2O, showing a complete dissolution at ~218 g/L. The solubility (g/L) was estimated while accounting for the polymer's volume contribution.

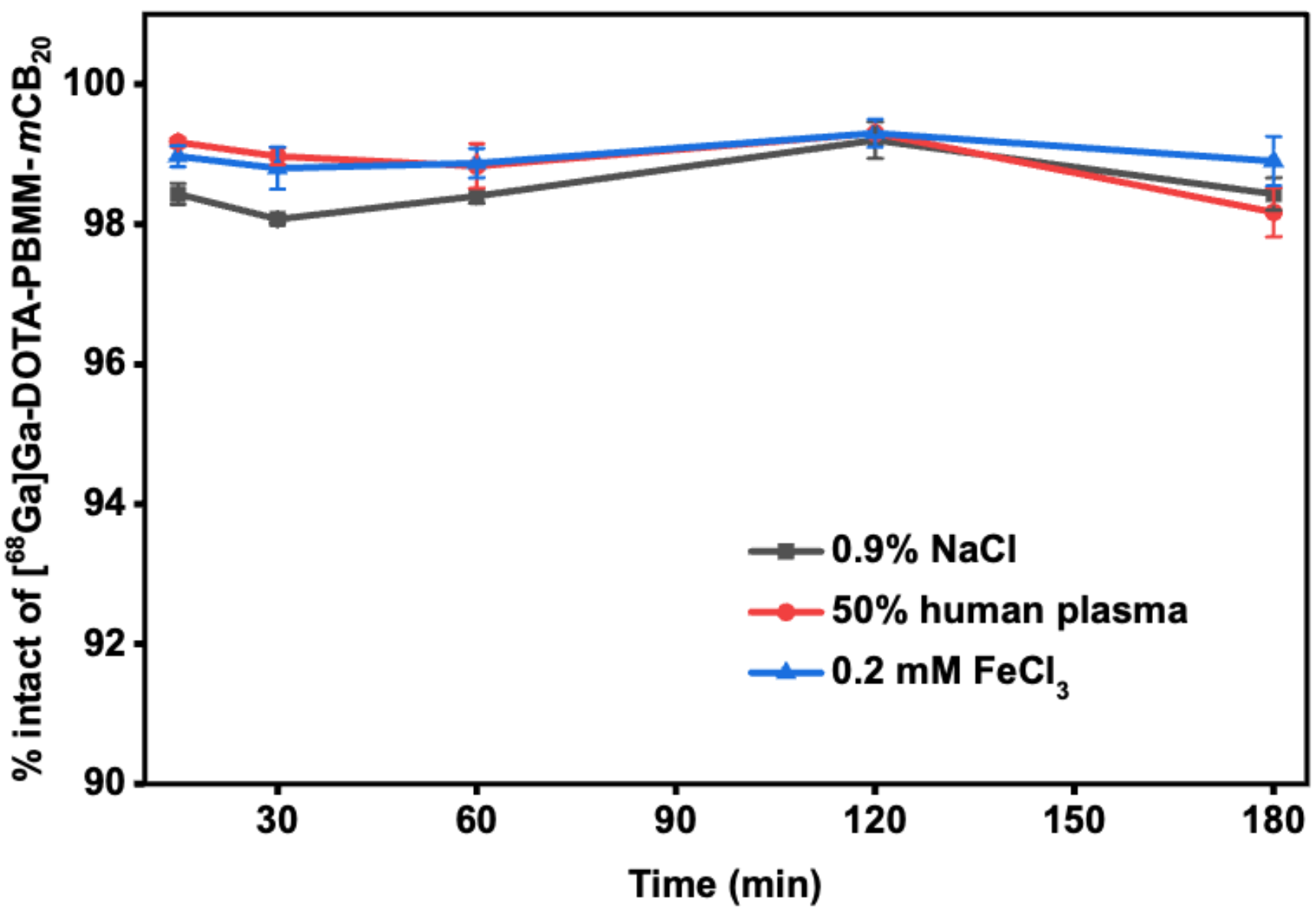

Figure S12. Radiolabel stability of [^{68}Ga]Ga-DOTA-PBMM-*m*CB_{20} in 0.9% NaCl (injection formulation), 50% human plasma, and 0.2 mM $FeCl_3$ over 180 of incubation at 37 °C (n = 3).

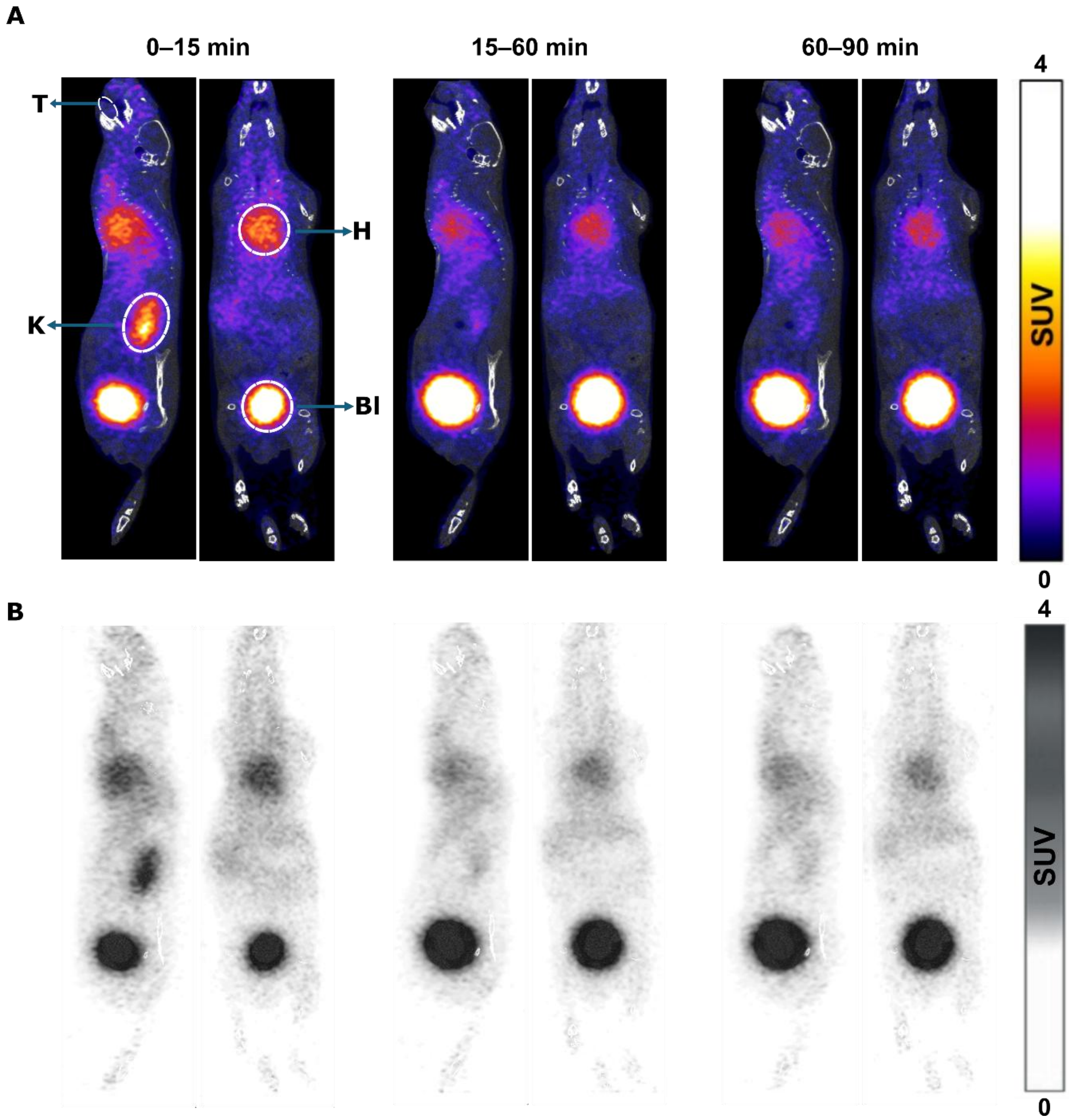

Figure S13. Representative dynamic PET images of [^{68}Ga]Ga-DOTA-PBMM-*m*CB_{20} in healthy mice summarized over three time windows (0–15 min, 15–60 min, and 60–90 min post-injection). (**A**) Sagittal (left) and coronal (right) PET/CT images for each interval, showing high blood pool activity in the heart (H), transient early renal activity in the kidneys (K), and progressive renal excretion to the bladder (Bl), with negligible accumulation in the healthy tongue (T). (**B**) Corresponding grayscale-rendered images of the same planes and time windows for improved contrast and visualization of the activity distribution.

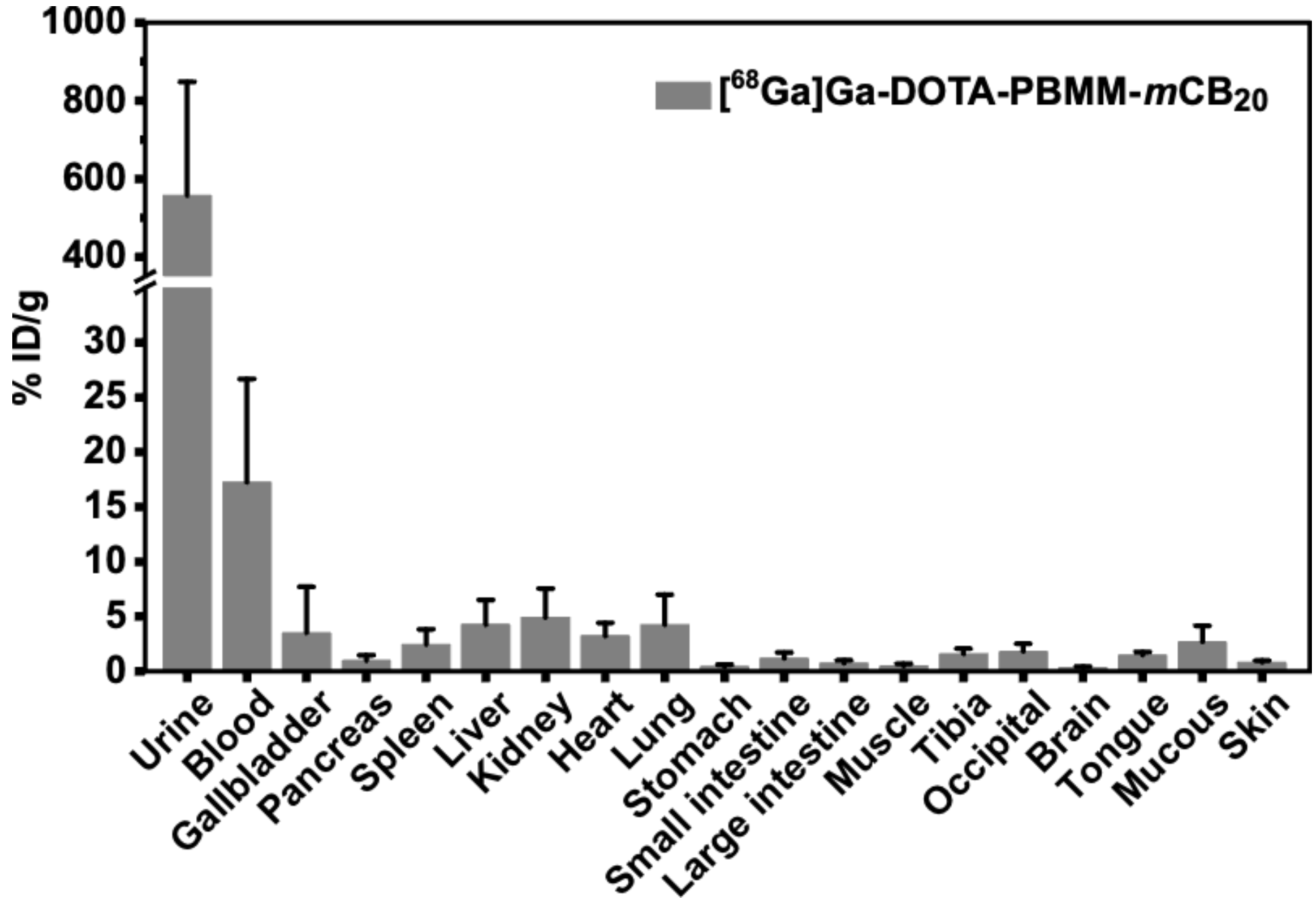

Figure S14. *Ex vivo* biodistribution of [^{68}Ga]Ga-DOTA-PBMM-*m*CB$_{20}$ at 90 min post-injection corroborating renal and urinary elimination and low off-target uptake in vital organs (n = 4).

Section 5: References

1. Menger, F. M.; Migulin, V. A. Synthesis and properties of multiarmed geminis. *J. Org. Chem.* **1999**, 64 (24), 8916-8921.
2. Lu, J.; Liang, L.; Weck, M. Micelle-based nanoreactors containing Ru-porphyrin for the epoxidation of terminal olefins in water. *J. Mol. Catal. A: Chem.* **2016**, 417, 122-125.
3. Lübtow, M. M.; Haider, M. S.; Kirsch, M.; Klisch, S.; Luxenhofer, R. Like dissolves like? A comprehensive evaluation of partial solubility parameters to predict polymer–drug compatibility in ultrahigh drug-loaded polymer micelles. *Biomacromolecules* **2019**, 20 (8), 3041-3056.
4. Agarwal, H. K.; Buszek, B.; Ricks, K. G.; Tjarks, W. Synthesis of closo-1,7-carboranyl alkyl amines. *Tetrahedron Lett.* **2011**, 52 (43), 5664-5667.
